# Alveolar progenitor differentiation and lactation depends on paracrine inhibition of notch via ROBO1/CTNNB1/JAG1

**DOI:** 10.1242/dev.199940

**Published:** 2021-11-11

**Authors:** Oscar Cazares, Sharmila Chatterjee, Pinky Lee, Catherine Strietzel, J. W. Bubolz, Gwyndolen Harburg, Jon Howard, Sol Katzman, Jeremy Sanford, Lindsay Hinck

**Affiliations:** 1 Institute for the Biology of Stem Cells, University of California, Santa Cruz, CA 95064, USA; 2 Department of Molecular, Cell and Developmental Biology, University of California, Santa Cruz, CA 95064, USA; 3 Department of Biomolecular Engineering, University of California, Santa Cruz, CA 95064, USA; 4 Zoetis Inc. 333 Portage Street, Building 300, Kalamazoo, MI 49007, USA

**Keywords:** Robo, Notch, Beta-catenin, Jagged1, Alveolar progenitor, Mammary gland, Mouse

## Abstract

In the mammary gland, how alveolar progenitor cells are recruited to fuel tissue growth with each estrus cycle and pregnancy remains poorly understood. Here, we identify a regulatory pathway that controls alveolar progenitor differentiation and lactation by governing Notch activation in mouse. Loss of *Robo1* in the mammary gland epithelium activates Notch signaling, which expands the alveolar progenitor cell population at the expense of alveolar differentiation, resulting in compromised lactation. ROBO1 is expressed in both luminal and basal cells, but loss of *Robo1* in basal cells results in the luminal differentiation defect. In the basal compartment, ROBO1 inhibits the expression of Notch ligand *Jag1* by regulating β-catenin (CTNNB1), which binds the *Jag1* promoter. Together, our studies reveal how ROBO1/CTTNB1/JAG1 signaling in the basal compartment exerts paracrine control of Notch signaling in the luminal compartment to regulate alveolar differentiation during pregnancy.

## Abstract (precis)

**Summary:** This study demonstrates that Robo1/β-catenin/JAG1 signaling in basal epithelial cells regulates luminal Notch activity and alveolar progenitor differentiation, offering a non-hormonal way to target milk production and mitigate lactation insufficiency.

## INTRODUCTION

The mammary gland (MG) is a distinguishing feature of mammals, with its ability to produce and secrete milk for nourishment of offspring. Throughout a female's reproductive life, this remarkable organ retains the ability to generate milk-producing alveoli, undergoing prodigious proliferation and differentiation of mammary epithelial cells with each pregnancy and, on a minor scale, every estrous cycle. The mammary epithelium generates a tree-like bi-layered ductal network, comprising an outer layer of myoepithelial cells that contract to expel milk, and an inner layer of luminal epithelial cells that either line the ducts (ductal epithelial cells) or generate milk during lactation (alveolar epithelial cells) (Macias and Hinck, 2012). Lineage labeling studies have demonstrated that enduring, lineage-restricted progenitor cells play a crucial role in generating the large number of luminal and basal cells required to build a milk supply (Fu et al., 2020). Yet, molecular mechanisms governing the cyclical expansion, differentiation and renewal of such lineage-restricted progenitors are still being discovered.

Paracrine interactions between the luminal and basal compartments have proven crucial at all stages of MG development, including during alveologenesis. Progesterone and prolactin initiate the alveolar switch that is subsequently controlled by factors such as Notch ligands that regulate alveolar progenitor cell (AVP) self-renewal, expansion and differentiation (Oakes et al., 2006). Studies have shown that Notch signaling must be downregulated to allow alveologenesis because this process is severely diminished by the overexpression of Notch receptor intracellular domains (ICDs) (Hu et al., 2006; Jhappan et al., 1992; Raafat et al., 2011; Smith et al., 1995). Sustained Notch activation also severely impairs alveologenesis in *Elf5−/−* mice, which have MGs containing a surplus of stem/progenitor cells (Chakrabarti et al., 2012; Choi et al., 2009). All four Notch receptors are expressed in temporally and spatially restricted patterns in subpopulations of basal and luminal mammary epithelial cells (Bach et al., 2017; Bouras et al., 2008; Raafat et al., 2011; Raouf et al., 2008), with lineage-tracing studies suggesting distinct roles for these receptors in directing stem/progenitor cell activity (Lafkas et al., 2013; Rodilla et al., 2015; Sale et al., 2013). By comparison, expression of Notch ligands (JAG1, JAG2 and DLL1) is largely confined to the basal compartment (Bach et al., 2017; Bouras et al., 2008; Raafat et al., 2011; Raouf et al., 2008; Xu et al., 2012). Thus, while there is an indisputably important role for Notch signaling in governing alveolar development, how Notch receptors are regulated in subsets of mammary progenitor cells by different Notch ligands remains unclear.

ROBOs are evolutionarily conserved receptors belonging to the immunoglobulin superfamily. ROBOs are canonically known for their role in cell and axon guidance via their regulation of the cytoskeleton, but in recent years our understanding of ROBO action has expanded considerably. We now know that ROBOs are essential to cell proliferation, survival and fate specification in numerous epithelial tissues (Ballard and Hinck, 2012). In stem/progenitor cells, ROBO signaling regulates the subcellular localization and therefore function of β-catenin (CTNNB1). For example, in the MG, ROBO2 signaling promotes stem cell senescence by inhibiting the nuclear localization of CTNNB1 and derepressing p16^INK4a^ (CDKN2A) (Harburg et al., 2014). In contrast, in the mammalian intestine, ROBO1 signaling enhances the nuclear localization of CTNNB1 and protects stem cells from radiation-induced injury (Zhou et al., 2013). These and other studies show that a major way ROBO signaling impacts WNT signaling is by governing the activation of CTNNB1 (Ballard and Hinck, 2012).

Wnt and Notch signaling pathways play crucial roles during development, particularly during the assignment of cell fate and subsequent expansion and differentiation of stem and progenitor cells (Hayward et al., 2008). In renewing tissues, how these linked signaling pathways control the deployment of lineage-restricted progenitor cells is still poorly understood. Here, we examined the consequences of *Robo1* loss in the mammary gland and found activated Notch signaling, reduced alveolar differentiation and compromised lactation. Using transplantation and organoid studies, we show that ROBO1 acts in the basal epithelial compartment to regulate the expression of JAG1 through CTNNB1. Our data support a model in which paracrine control of Notch activity in the luminal compartment by ROBO1/CTNNB1/JAG1 in the basal compartment governs alveolar progenitor expansion and differentiation into milk-producing alveoli.

## RESULTS

### Loss of *Robo1* diminishes alveologenesis and lactogenesis

**Fig. 1.**
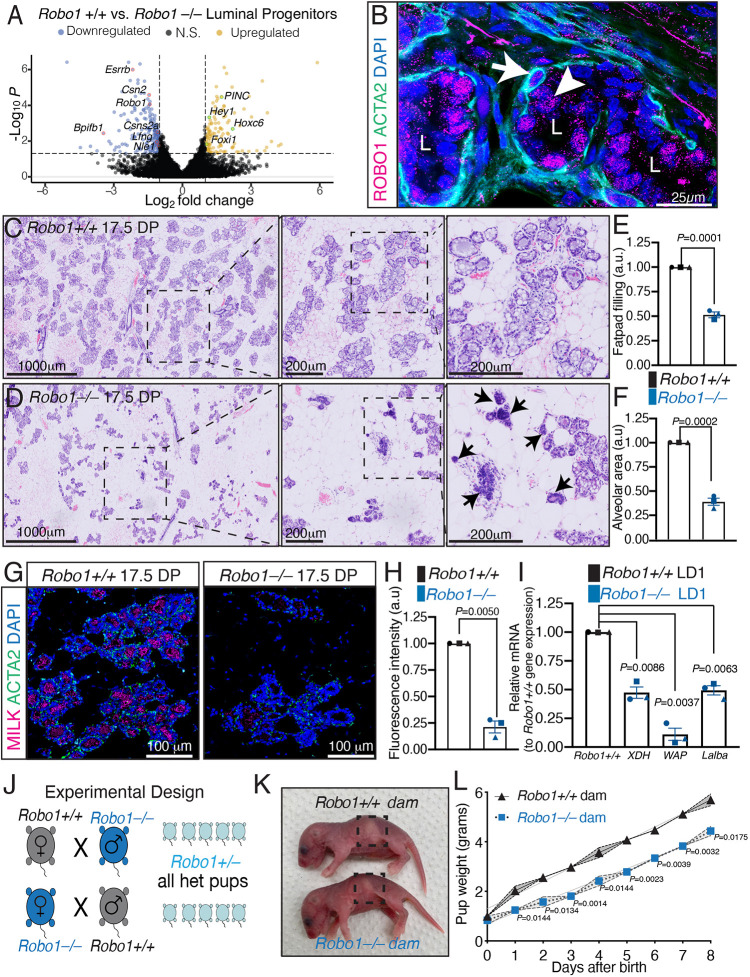
**Loss of *Robo1* diminishes alveologenesis and lactogenesis.** (A) Volcano plot of significantly altered mRNAs involved in alveologenesis. (B) Representative confocal image of CUBIC cleared alveoli from 7.5 DP *Robo1+/+* tissue shows ROBO1 (magenta; white arrow) with basal marker smooth muscle actin (ACTA2; green), and ROBO1 (magenta; white arrowhead) in underlying luminal cells. (C-F) Representative H&E-stained whole-mount sections of 17.5 DP *Robo1+/+* (C) and *Robo1−/−* (D) littermates. Insets show magnified images of boxed areas. Arrows identify compact *Robo1−/−* alveoli (D). Quantification of fat pad filling (E) and average alveolar size (F) show reduced *Robo1−/−* alveologenesis. (G,H) Representative confocal images (G) and quantification (H) show reduced milk (magenta) with ACTA2 (green) in 17.5 DP *Robo1−/−* MGs. (I) RT-qPCR on lactation day 1 (LD1) *Robo1−/−* MGs shows reduced milk protein gene expression: whey acidic protein (*WAP*), alpha-lactalbumin (*Lalba*) and xanthine dehydrogenase (*XDH*). (J) Mating strategy to measure milk production. (K) Images of pups at LD1, boxed area shows reduced stomach milk in pup fed by *Robo1−/−* dam. (L) Quantification shows pups fed by *Robo1−/−* dam gain less weight (two-way ANOVA followed by a two-tailed, unpaired *t*-test). *n*=3 independent experiments, five images/n E,F,H,I. Data are represented as mean±s.e.m. Statistical analysis was performed using a two-tailed, unpaired Student's *t*-test with Welch's correction or as stated above. N.S., not significant. See also Fig. S1.

To identify cellular processes that may be regulated by ROBO1, we performed fluorescence-activated cell sorting (FACS) to purify populations of cells harvested from wild-type (WT) and *Robo1^tm1Matl/tm1Matl^* (herein referred to as *Robo1−/−* or KO) mature, virgin MGs: basal cells (Lin^–^CD24^+^CD29^hi^; BC), mature luminal cells (Lin^–^CD24^lo^CD29^+^CD61^–^; ML), and luminal progenitor cells (Lin^–^CD24^lo^CD29^+^CD61^+^; LP) (Harburg et al., 2014). We then performed RNA-seq analysis. Piquing our interest was the KEGG analysis on LPs that revealed not only pathways consistent with current data on ROBO function, such as extracellular matrix receptor interaction and regulation of actin cytoskeleton (Fig. S1A,B) (Blockus and Chedotal, 2016), but also downregulation of Jak-STAT and prolactin signaling pathways, which could interfere with successful alveologenesis in pregnant *Robo1−/−* animals. We also observed downregulation of genes involved in the terminal differentiation of alveolar epithelium, including: estrogen related receptor beta (*Esrrb*); BPI fold containing family B member 1 (*Bpifb1*), a transcription factor that activates milk protein gene expression; milk protein genes *Csn2* and α-casein (*Csn1*; also known as *Csn1s1*); and lunatic fringe (*Lfng*), a glycosyltransferase that regulates Notch signaling (Fig. 1A) (Forster et al., 2002; Hicks et al., 2000; Pegolo et al., 2018). Concordantly, upregulated genes included: a luminal progenitor cell marker, *Foxi1*; a long non-coding RNA, *Pinc*, which inhibits terminal differentiation of alveolar cells by activating Notch signaling; and *Hey1*, a downstream effector of Notch signaling (Fig. 1A) (Pellacani et al., 2019; Shore et al., 2012).

To investigate a putative role for ROBO1 during pregnancy, we evaluated its gene expression in whole MGs using RT-qPCR and observed a peak in its expression at 7.5 day pregnancy (DP) (Fig. S1C). Interrogation of a single cell RNA-seq dataset identified *Robo1* as highly expressed across the basal compartment with lower expression in hormone-sensing luminal cells (Fig. S1C,D) (Bach et al., 2017). We performed immunohistochemistry (IHC) on thick sections of CUBIC-cleared 7.5 DP tissue and observed ROBO1 in subpopulations of myoepithelial and luminal cells of tertiary buds (Fig. 1B, Movie 1), with no expression observed in *Robo1−/−* tissue (Fig. S1F,G) (Long et al., 2004).

To explore the potential role of ROBO1 during alveolar development, we examined the phenotype of *Robo1−/−* mice. Histological analysis of whole mounted (Fig. S1H,I) and sectioned (Fig. 1C,D) 17.5 DP MGs from *Robo1*+/+ and *Robo1*−/− littermates showed a reduction (over 50%) in both *Robo1−/−* epithelial fat pad filling and alveolar area, with *Robo1−/−* alveoli appearing compact and lumenless (Fig. 1E,F). EdU labeling at mid-pregnancy (10.5 DP) showed reduced proliferation of epithelial cells in *Robo1−/−* tissue, consistent with the observed reduction in alveolar development (Fig. S1J-L). IHC on 17.5 DP sections with anti-milk antibody revealed an ∼80% decrease in milk protein expression in *Robo1*−/− tissue (Fig. 1G,H) and RT-qPCR at lactation day (LD) 1 also showed decreased expression of milk protein genes in *Robo1−/−* MGs (Fig. 1I). To assess functional consequences of this reduced alveolar development, we measured the ability of *Robo1*+/+ and *Robo1*−/− dams to support the growth of their first litter by measuring the weight of their heterozygous pups (Fig. 1J). Heterozygous pups nursed by *Robo1−/−* dams contained less stomach milk and weighed significantly less compared with heterozygous pups nursed by *Robo1*+/+ dams (Fig. 1K,L). Thus, loss of *Robo1* caused a deficiency in alveologenesis during pregnancy and milk production during lactation.

### ROBO1 regulation of mammary alveologenesis is intrinsic to the epithelium

**Fig. 2.**
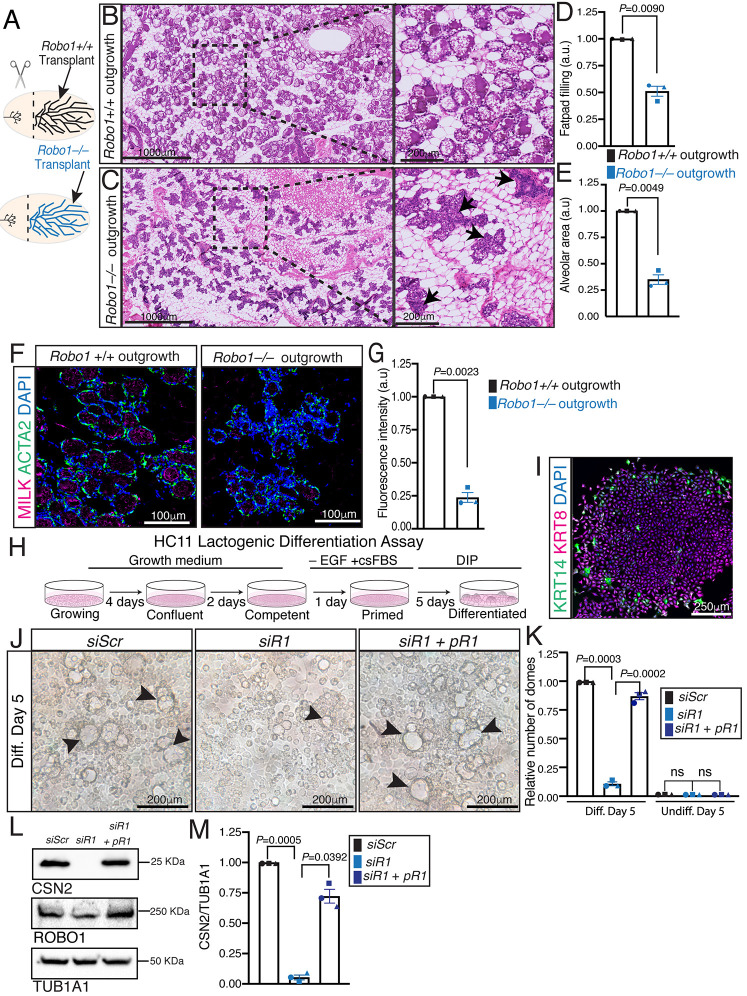
**ROBO1 regulation of mammary alveologenesis is intrinsic to epithelium.** (A) Diagram of transplantation. (B,C) Representative H&E whole-mount sections of 17.5DP *Robo1+/+* (B) and *Robo1−/−* (C) contralateral outgrowths at 17.5 DP. Insets are magnified images of boxed areas. Arrows identify compact *Robo1−/−* alveoli. (D,E) Quantification of fat pad filling (D) and average alveolar size (E) show reduced *Robo1−/−* alveologenesis. (F,G). Representative confocal images (F) and quantification (G) show reduced milk (magenta) with ACTA2 (green) in 17.5 DP *Robo1−/−* outgrowths. (H) Schematic of the stages of HC11 lactogenic differentiation. (I) Representative confocal image of undifferentiated HC11 cells shows expression of KRT14+ (green) cells encircling KRT8+ (magenta) cell. (J,K) Differential interference contrast (DIC) images (J) and quantification (K) of *siScr* and *Robo1* KD in differentiated HC11 cells show reduced milk dome formation that is largely rescued by *Robo1* overexpression. Arrowheads identify domes. (L,M) Immunoblot (L) and quantification (M) show reduced CSN2 expression that is largely rescued by *Robo1* overexpression (two-tailed paired *t*-test). *n*=3 independent experiments, 10 images/*n* (D,E,G,K). Data are represented as mean±s.e.m. Statistical analysis was performed using a two-tailed, unpaired Student's *t*-test with Welch's correction or as stated above. ns, not significant. See also Fig. S2.

To test whether the observed defect in alveologenesis was due to *Robo1* loss in the mammary epithelium, and not due to its global deletion in the animal, we performed transplantation assays. *Robo1+/+* and *Robo1−/−* littermate tissue fragments were contralaterally transplanted into *Foxn1^nu/nu^* host mice pre-cleared of their endogenous mammary parenchyma (Fig. 2A). Allowing ten weeks for epithelial outgrowth, the animals were then mated and MG tissue harvested on 17.5 DP. Histological analysis of whole-mounted (Fig. S2A,B) and sectioned (Fig. 2B,C) MG outgrowths showed reduced epithelial fat pad filling (∼50%) and reduced alveolar area in *Robo1−/−* outgrowths (Fig. 2D,E), similar to the reductions observed in the intact *Robo1−/−* MG (Fig. 1E,F). This reduction in alveolar development was also accompanied by reduced expression of both milk (Fig. 2F,G) and the lipid binding protein perilipin 2 (PLIN2) (Fig. S2C,D).

To further assess the epithelial intrinsicality of the *Robo1*−/− phenotype, we turned to HC11 cells, a well-established prolactin-sensitive lactation model that undergoes a step-wise differentiation process (Fig. 2H) (Ball et al., 1988; Desrivieres et al., 2003). This heterogeneous cell line grows with keratin 14 (KRT14)-positive basal cells encircling keratin 8 (KRT8)-positive luminal cells (Fig. 2I). We knocked down *Robo1* and differentiated these cells (Fig. S2E,F), observing fewer milk domes and reduced CSN2 compared with control (*siScr*) knockdown (KD); these effects were rescued by overexpression of siRNA resistant p*Robo1* (Fig. 2J-M). No milk domes were formed in *shScr* or *Robo1* KD undifferentiated cells (Fig. S2G). Collectively, these data revealed that loss of *Robo1* in the epithelial compartment of the MG reduces alveolar development and differentiation.

### ROBO1 regulates notch signaling in HC11 cells and luminal progenitors

**Fig. 3.**
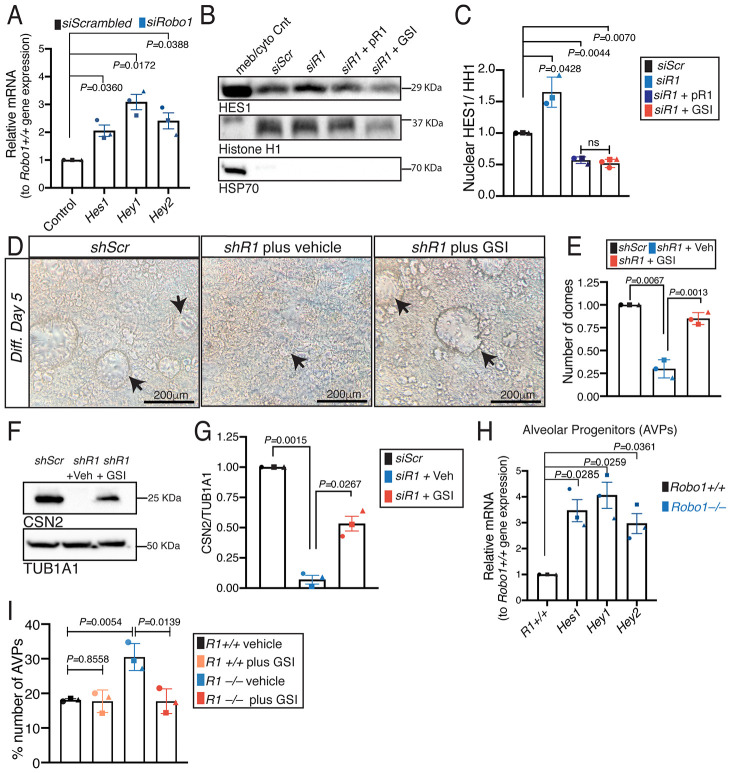
**ROBO1 regulates notch signaling in luminal progenitors and HC11 cells.** (A) RT-qPCR on *siScr* and *Robo1* (*siR1*) KD primed HC11 cells shows increased Notch effector expression with loss of *Robo1*. (B,C) Immunoblot (B) and quantification (C) on the nuclear fraction of *siScr* and *Robo1* KD primed HC11 cells show increased nuclear HES1 with *Robo1* loss and rescue by either *Robo1* overexpression or GSI treatment (two-tailed paired *t*-test). (D,E) HC11 dome assay (D) and quantification (E) show fewer domes with *Robo1* KD and rescue with GSI treatment. Arrows identify domes. (F,G) CSN2 immunoblot (F) and quantification (G) show decreased milk production with *Robo1* KD and partial rescue by GSI treatment (two-tailed paired *t*-test). (H) RT-qPCR on FACS-purified *Robo1+/+* and *Robo1−/−* AVPs shows increased Notch effector expression with loss of *Robo1*. (I) FACS quantification of the AVP subpopulation shows more AVPs in *Robo1*−/− MGs and rescue to *Robo1+/+* levels by GSI treatment. *n*=3 independent experiments, five images/*n*. Data are represented as mean±s.e.m. Statistical analysis was performed using a one-way ANOVA followed by a two-tailed, unpaired Student's *t*-test with Welch's correction or as indicated above. ns, not significant. See also Fig. S3.

Previous studies have shown that downregulation of Notch signaling in the luminal compartment is required for successful alveologenesis (Chakrabarti et al., 2012; Choi et al., 2009; Hu et al., 2006; Jhappan et al., 1992; Smith et al., 1995). Our RNA-seq data indicated that Notch signaling is upregulated in virgin *Robo1−/−* LPs (Fig. 1A). To further investigate, we assayed the expression of Notch effectors in confluent *shScr* and *Robo1* KD HC11 cells and observed upregulation of *Hes1*, *Hey1* and *Hey2* by RT-qPCR (Fig. 3A). We also observed increased HES1 in the nuclear fraction of *Robo1* KD HC11 cell lysates (Fig. 3B,C). As controls, we overexpressed p*Robo1* in KD cells and also treated KD cells with gamma secretase inhibitor (GSI) RO4929097, and found that both treatments not only prevented the increase in nuclear HES1, but also reduced its levels to lower than control (Fig. 3B,C). We also examined HES1 expression by intracellular flow immunostaining (Fig. S3A). As a positive control, we treated HC11 cells with JAG1 peptide and observed increased nuclear HES1 (Fig. S3A). KD of *Robo1* also increased the nuclear HES1 signal, an effect that was dampened by GSI (Fig. S3A). Next, we assessed whether Notch inhibition could rescue the *Robo1* KD HC11 phenotype by treating *shScr* and *Robo1* KD cells for 24 h at priming with either vehicle or GSI, followed by differentiation. As shown previously (Fig. 2J,K), *Robo1* KD resulted in fewer milk domes and decreased CSN2, effects that were rescued by GSI treatment (Fig. 3D-G).

Across a wide array of tissues, Notch signaling regulates stem/progenitor cells, coordinating their self-renewal, expansion and differentiation. To examine whether ROBO1 regulates Notch signaling in mammary progenitor cells, we performed RT-qPCR on Notch effectors, focusing on the FACS-purified AVP subpopulation (CD14+;cKit^−/lo^) (Fig. 3H, Fig. S3B) (Asselin-Labat et al., 2011; Shore et al., 2012). The expression of *Hes1*, *Hey1* and *Hey2* were all increased in *Robo1−/−* AVPs (Fig. 3H). To assess whether this upregulation of Notch signaling in *Robo1*−/− animals affected the number of progenitor cells, we FACS-analyzed luminal subpopulations from MGs of nulliparous *Robo1+/+* and *Robo1−/−* littermates and found that *Robo1*−/− MGs contained significantly more AVPs, fewer LPs (trending) and no difference in the number of mature luminal cells (MLs) (Fig. S3C). Next, we orally treated mice with GSI (10 mg/kg) or vehicle daily for 5 days before harvesting the MGs and FACS-purifying subpopulations. GSI-treatment of *Robo1−/−* animals decreased the number of AVPs to WT levels and reduced the expression of Notch effectors *Hey1* and *Hes1* (Fig. 3I, Fig. S3D). GSI treatment of *Robo1+/+* animals did not affect AVP number (Fig. 3I), and it had no effect on *Hey1* expression, but did reduce *Hes1* (Fig. S3D). Collectively, these data indicated that ROBO1 promotes alveolar development by inhibiting Notch signaling, and thereby restricting AVP renewal and expansion.

### ROBO1 regulates luminal notch signaling from the basal compartment

**Fig. 4.**
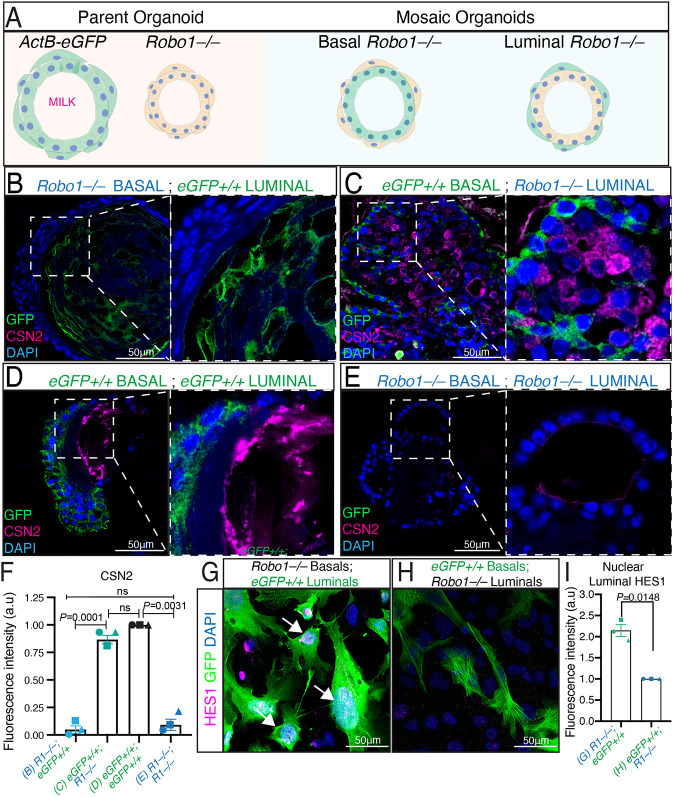
**ROBO1 regulates luminal Notch signaling from the basal compartment.** (A) Diagram showing mosaic organoids that contain *Robo1−/−* (orange) basal or luminal cells combined with *ACTb-eGFP Robo1+/+* (green: *eGFP+/+*) basal or luminal cells. (B-F) Representative confocal images (B-E) and quantification (F) of paraffin-embedded organoid sections immunostained for GFP (green) and milk protein CSN2 (magenta). Organoids with *Robo1−/−* basal cells (KO/WT) and (KO/KO) show little CSN2 staining (B,E,F), whereas organoids with *GFP+/+* basal cells (WT/KO) and (WT/WT) show robust CSN2 staining (C,D,F). (G-I) Representative ICC (G,H) and quantification (I) of *Robo1−/−* basal cells co-cultured with *eGFP+/+* luminal cells show increased nuclear HES1 in the *eGFP+/+* luminal cells (arrows) (G,I). In contrast, co-cultures of *eGFP+/+* basal cells with *Robo1−/−* luminal cells show little or no nuclear HES1 in the *Robo1−/−* luminal cells (H,I). *n*=3 independent experiments. Data are represented as mean±s.e.m. Statistical analysis was performed using a one-way ANOVA followed by a two-tailed, unpaired Student's *t*-test with Welch's correction. ns, not significant. See also Fig. S4.

ROBO1 is expressed in both luminal and basal cells of the MG (Fig. 1B, Fig. S1D,E). To determine whether ROBO1 promotes alveolar differentiation through its action specifically in one of these cell types, we generated organoids that are mosaic for *Robo1* expression (Fig. 4A) (Rubio et al., 2020). *Robo1−/−* basal cells were mixed with *Robo1+/+* luminal cells (KO/WT) and, vice versa, *Robo1+/+* basal cells were mixed with *Robo1−/−* luminal cells (WT/KO) (Fig. 4B,C). Cells from *ACTb-EGFP* (*eGFP+/+*) mice were used to distinguish WT from KO cells and, as controls, WT/WT and KO/KO organoids were also generated (Fig. 4D,E). Organoids were cultured in Matrigel, followed by differentiation in prolactin-containing media for 5 days (Fig. S4A). The sectioned organoids were immunostained for GFP along with CSN2 (Fig. 4B-E) or the basal marker KRT14 (Fig. S4B). When *Robo1*−/− basal cells were mixed with *GFP+/+* luminal cells (KO/WT), the resulting mosaic organoids produced little/no milk upon differentiation, suggesting that ROBO1 in basal cells functions to enhance luminal differentiation (Fig. 4B,F). Supporting this notion was the observation that when WT basal cells were mixed with *Robo1*−/− luminal cells (WT/KO), the resulting organoids displayed robust CSN2 staining upon differentiation (Fig. 4C,F). This robust production of the CSN2 milk protein was also observed in WT/WT organoids (Fig. 4D,F), whereas KO/KO organoids were similar to KO/WT organoids and produced little or no CSN2 (Fig. 4E,F). Next, we co-cultured *Robo1−/−* basal cells with *eGFP+/+* luminal cells and observed increased nuclear HES1 in the *eGFP+/+* luminal cells adjacent to *Robo1−/−* basal cells (Fig. 4G,I). As a control, we performed the reverse experiment and co-cultured *eGFP+/+* basal cells with *Robo1−/−* luminal cells and observed little or no expression of nuclear HES1 in the *Robo1−/−* luminal cells (Fig. 4H,I). Together, these data showed that ROBO1 was required in mammary basal cells where it functions to repress luminal Notch signaling and support milk production upon hormonal stimulation.

### ROBO1 inhibits *Jag1* expression in basal cells via CTNNB1

**Fig. 5.**
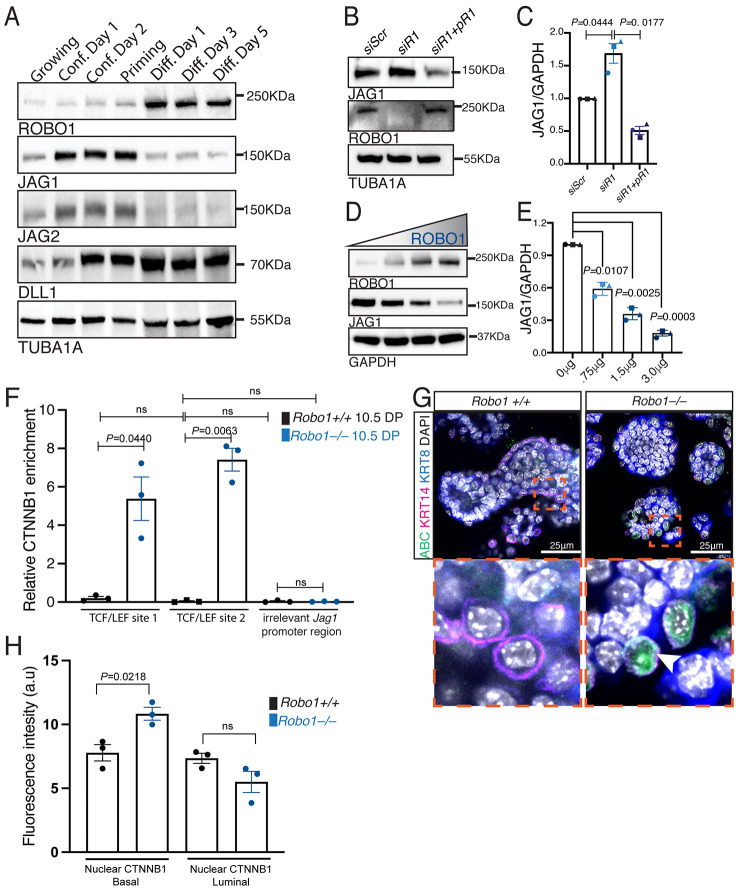
**ROBO1 inhibits JAG1 expression in basal cells via CTTNB1.** (A) Immunoblot shows the inverse regulation of ROBO1 with respect to JAG1 and JAG2 and no change in DLL1 over HC11 differentiation. (B,C) Immunoblot (B) and quantification (C) show increased JAG1 with *Robo1* KD and rescue with *Robo1* overexpression (two-tailed paired *t*-test). (D,E) Immunoblot (D) and quantification (E) show that increasing overexpression of *Robo1* results in decreasing JAG1 (two-tailed, paired Student's *t*-test). (F) RT-qPCR using primers within the *Jag1* promoter either specific to two Tcf/Lef binding sites or to an irrelevant location (control) shows increased CTNNB1 chromatin immunoprecipitation in FACS-purified 10.5 DP *Robo1*−/− basal cells. (G,H) Representative 3D confocal images (G) and quantification (H) of differentiated *Robo1+/+* and *Robo1−/−* organoids show increased nuclear CTNNB1 (green) staining in *Robo1−/−* KRT14+ (magenta) basal cells. Bottom panels in G show magnification of boxed areas in top panels. *n*=3 independent experiments. Data are represented as mean±s.e.m. Statistical analysis was performed using a one-way ANOVA followed by a two-tailed, unpaired Student's *t*-test with Welch's correction or as stated above. ns, not significant. See also Fig. S5.

Notch ligands regulate the activation of Notch receptors. Examination of a single cell RNA-seq dataset revealed *Jag1*, *Jag2* and *Dll1* expression in basal mammary epithelial cells, and very little to no expression of *Dll3* and *Dll4* (Fig. S5A-E) (Bach et al., 2017). We examined the expression of JAG1, JAG2 and DLL1 over the course of HC11 differentiation and observed inverse regulation with respect to ROBO1, with high levels of JAG1/JAG2 during competence and priming but high levels of ROBO1 during differentiation (Fig. 5A). In contrast, DLL1 increases from confluence through differentiation (Fig. 5A). To determine whether ROBO1 regulates the expression of Notch ligands, we performed KD of *Robo1* in HC11 cells and observed by immunoblot increased JAG1 that is not only rescued but also further decreased by p*Robo1* overexpression (Fig. 5B,C); we detected no changes in JAG2 or DLL1 expression (Fig. S5F,G). We also examined JAG expression in FACS-purified populations of *Robo1+/+* and *Robo1−/−* basal cells and found more JAG1 in KO, compared with WT, cells but no significant change in JAG2 (Fig. S5H-J). To further explore, we overexpressed increasing amounts of *Robo1* in HEK293 cells and detected a dose-dependent decrease in JAG1 (Fig. 5D,E). Previous studies showed that the transcriptional function of CTNNB1 is activated in *Robo1*−/− basal epithelial cells (Macias et al., 2011). To determine whether *Jag1* is a target of CTNNB1 in the MG, we performed a Cut&Run ChIP assay using anti-CTNNB1 antibody on *Robo1*+/+ and *Robo1*−/− FACS-purified 7.5 DP basal mammary epithelial cells (CD49f^hi^;CD24+). ChIP PCR fragments were amplified using primers that were specific for two different Tcf/Lef binding sites upstream of the *Jag1* promoter along with control primers that recognized an irrelevant region of this promoter (Fig. 5F). RT-qPCR analysis of these regions revealed a significant increase in CTNNB1 occupancy at both binding sites in *Robo1−/−* basal mammary epithelial cells (Fig. 5F, Fig. S5K). Next, we generated and cultured *Robo1+/+* and *Robo1−/−* organoids in Matrigel, followed by differentiation in prolactin-containing media for 5 days. The organoids were immunostained for basal and luminal markers KRT14 and KRT8, respectively, and an antibody directed against activated nuclear CTNNB1 (Staal et al., 2002) (Fig. 5G). We observed more nuclear expression of CTNNB1 in *Robo1−/−* basal cells and no significant change in luminal cells (Fig. 5H). Together, these data showed that ROBO1 represses luminal Notch signaling by inhibiting the nuclear localization of CTNNB1 in basal cells, thereby preventing CTNNB1 from directly enhancing the expression of Notch ligand JAG1.

### JAG1 in basal cells inhibits luminal differentiation and milk production

**Fig. 6.**
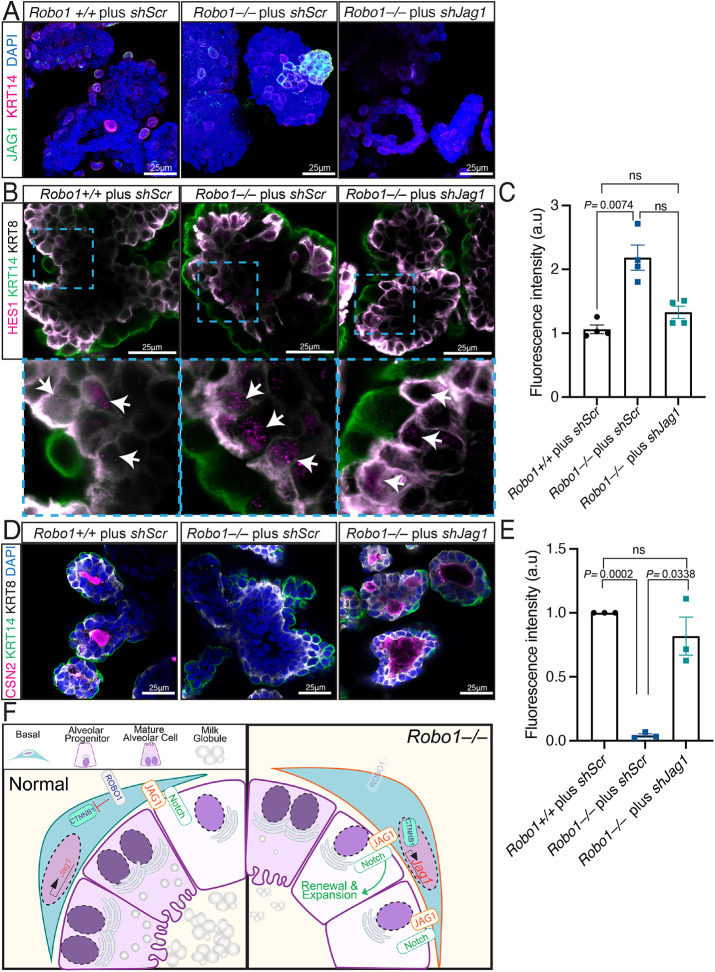
**JAG1 in basal cells inhibits luminal differentiation and milk production.** (A) Representative 3D confocal images of *Robo1+/+* and *Robo1−/−* organoids infected with either *shScr* or *shJag1* and stained for JAG1 (green) and KRT14 (magenta) show little JAG1 in *Robo1+/+* basal cells, and an increase in JAG1 staining in *Robo1−/−* basal cells that is decreased by KD of *Jag1* (*shJag1*). (B,C) Representative 3D confocal images (B) and quantification (C) of *Robo1+/+* and *Robo1−/−* organoids infected with either *shScr* or *shJag1* and stained for HES1 (magenta) and KRT14 (green) show little HES1 in *Robo1+/+* basal cells, and an increase in HES1 expression in *Robo1−/−* basal cells that is decreased by KD of *Jag1* (*shJag1*). Bottom panels in B show magnification of boxed areas in top panels. Arrows indicate nuclear HES1. (D,E) Representative 3D confocal images (D) and quantification (E) of *Robo1+/+* and *Robo1−/−* organoids infected with either *shScr* or *shJag1* and stained for CSN2 (magenta) and KRT14 (green) show robust CSN2 expression in *Robo1+/+* organoids and in *Robo1−/−* organoids with *Jag1* (*shJag1*) KD; there is little or no CSN2 staining in *Robo1−/−* (*shScr*) organoids. (F) Schematic of our model showing how JAG1 expression is held in check in the cytoplasm in wild-type (left) basal cells of mid-pregnant MGs by ROBO1 inhibition of CTNNB1, thereby promoting differentiation. In contrast in *Robo1−/−* (right) basal cells, nuclear CTNNB1 enhances *Jag1* expression and JAG1/Notch signaling inhibits differentiation while promoting alveolar progenitor renewal and expansion. *n*=3 independent experiments minimum. Statistical analysis was performed using a one-way ANOVA followed by a two-tailed, unpaired Student's *t*-test with Welch's correction. ns, not significant. See also Fig. S6.

Although many studies have demonstrated the importance of Notch receptor regulation in the MG during pregnancy (Hu et al., 2006; Jhappan et al., 1992; Smith et al., 1995), much less is known about the role of Notch ligands. To further probe the role of JAG1 in mammary alveologenesis, we overexpressed *Jag1* (*pJag1*) and then differentiated HC11 cells, observing reduced milk dome formation (Fig. S6A,B). In contrast, when *Jag1* was knocked down in cells followed by differentiation, dome formation was enhanced (Fig. S6A,B). Next, we used lentiviral infection to KD *Jag1* (*shJag1*) in *Robo1*−/− primary cells. Organoids were then generated and cultured in Matrigel, followed by differentiation in prolactin-containing media for 5 days. The organoids were immunostained for basal and luminal markers KRT14 and KRT8, respectively, along with JAG1 to evaluate its expression, HES1 to evaluate Notch activity in the luminal compartment, and CSN2 to determine the extent of organoid differentiation (Fig. 6A-E). We observed little JAG1 and HES1 expression in the basal cells of *Robo1+/+* organoids infected with control *shScr* (Fig. 6A-C). As expected, these organoids displayed lumenal CSN2 accumulation, indicating robust differentiation (Fig. 6D,E). In contrast, JAG1 and nuclear HES1 expression were upregulated in *Robo1*−/− organoids infected with control *shScr* (Fig. 6A-C), and there was no detectable CSN2 immunostaining (Fig. 6D,E), indicating that the luminal cells of these organoids did not differentiate into milk-producing alveolar cells. However, KD of *Jag1* reduced both JAG1 and HES1 expression (Fig. 6A-C), and rescued the differentiation of *Robo1−/−* organoids as shown by CSN2 expression (Fig. 6D,E). Altogether, our data suggested that JAG1 is a key regulator of lactogenic differentiation and that downregulation of *Jag1* by ROBO1/CTNNB1 in the basal compartment inhibits luminal Notch activity, thereby promoting alveolar cell differentiation and CSN2 expression (Fig. 6F).

## DISCUSSION

Breastfeeding confers a host of lifelong benefits to both mother and child. For women, epidemiological studies show a significant decrease in breast and ovarian cancer risk with increased breastfeeding duration (Chowdhury et al., 2015). For children, there is substantial evidence that mother's milk is optimal nutrition that boosts immunity and diversifies the gut microbiome (Victora et al., 2016). Lactation insufficiency is the inability of a nursing individual to generate the daily volume of breast milk required to fully meet the nutritional needs of an infant. It is estimated that 23% to 64% of women worldwide experience lactation insufficiency, leading to weaning before 6 months, with primary lactation insufficiency due to inadequate glandular tissue accounting for 5% (Li et al., 2005; Sultana and Rahman, 2013; Taqi, 2014). Previous efforts to enhance milk production focused on the prolactin pathway and even drugs (approved in some countries, but not the USA), such as dopamine antagonist domperidone, are rarely prescribed due to negative side effects (Sewell et al., 2017). Consequently, the barriers to successful breastfeeding remain high, in part because there is still much unknown about the molecular underpinnings of lactation physiology, specifically how stem/progenitor cells are recruited to contribute to each pregnancy. Here, we identify a mechanism regulating the expansion and differentiation of AVPs into milk-producing alveolar cells.

Paracrine signaling between tissue compartments is a key mechanism guiding organ development. In the MG gland, we find that ROBO1 signaling in the basal compartment inhibits Notch activation in the luminal compartment by restricting the expression of *Jag1.* Our data show that, in the absence of *Robo1*, luminal Notch signaling through *Hes1*, *Hey1* and *Hey2* is increased, favoring AVP renewal and expansion at the expense of differentiation, a phenotype that is reversed by inhibiting Notch signaling. This is consistent with previous data demonstrating that overexpression of Notch-ICDs during pregnancy severely curtails mammary alveolar differentiation (Chakrabarti et al., 2012; Choi et al., 2009; Hu et al., 2006; Jhappan et al., 1992; Smith et al., 1995). Indeed, continued activation of Notch through ICD overexpression results in mammary hyperplasias and tumors (Hu et al., 2006; Jhappan et al., 1992), and the absence of *Robo1* also results in mammary hyperplasias (Marlow et al., 2008). The observation that ROBO1 signaling determines the level of Notch activity, and therefore the fate of daughter cells, has been demonstrated in the developing neocortex (Cardenas et al., 2018). ROBOs govern the balance between direct and indirect neurogenesis by regulating the expression levels of different Notch ligands. This, in turn, determines the size and complexity of the cerebral cortex and over evolutionary time has been responsible for increasing the circuitry of the mammalian brain (Cardenas et al., 2018). In the cerebral cortex, however, the signaling downstream of ROBO that leads to Notch ligand regulation has not been delineated.

There have been numerous reports that ROBO signaling regulates the activity of CTNNB1 through the PI3 kinase/Akt/GSK3-β pathway (Macias et al., 2011; Prasad et al., 2008; Shi et al., 2014; Tseng et al., 2010). Here, we demonstrate that during alveolar development loss of *Robo1* in basal mammary epithelial cells increases the association of CTNNB1 with the *Jag1* promoter, increasing *Jag1* expression. This is similar to the epidermis in which CTNNB1 also upregulates *Jag1*, activating Notch signaling, and in this context promoting the differentiation of hair follicle lineages (Estrach et al., 2006). In contrast, in the MG Notch activation in the luminal compartment during pregnancy inhibits alveolar differentiation (Chakrabarti et al., 2012; Choi et al., 2009; Hu et al., 2006; Jhappan et al., 1992; Smith et al., 1995), and our data suggest this is due to enhanced AVP renewal and expansion.

Regulated interplay between Notch and β-catenin (Wnt) signaling has been observed in many developmental contexts and is termed Wntch signaling to reflect the reciprocal and regulated roles these pathways play in determining the fate of cells (Munoz-Descalzo et al., 2012). In the MG, mounting evidence supports the role of lineage-restricted progenitor cells in generating the immense tissue growth and cell differentiation occurring with puberty, each pregnancy and every estrus cycle, raising the question of how unipotent precursor cells fuel this prodigious postnatal growth (Lilja et al., 2018; Lloyd-Lewis et al., 2018; Wuidart et al., 2016). Overexpression studies show that Notch signaling promotes the maintenance of progenitors and inhibits alveologenesis (Hu et al., 2006; Jhappan et al., 1992; Smith et al., 1995), whereas Wnt signaling supports progenitor expansion and differentiation (Imbert et al., 2001; Teuliere et al., 2005). Constitutive activation of CTNNB1, via overexpression of a truncated nuclear-localized form, results in precocious alveolar development, including the expression of milk protein genes (Imbert et al., 2001; Teuliere et al., 2005). These non-physiological model systems give insight into how Wnt and Notch signaling influence the probability of progenitor cells renewing, expanding or differentiating. Our data suggest that by 7.5 DP ROBO1-directed repression of nuclear CTNNB1 reduces *Jag1* expression in basal myoepithelial cells, triggering a switch that dials back Notch signaling in adjacent luminal cells to promote the differentiation of AVPs. ROBO signaling governs this switch, allowing the cross-regulation of Notch and Wnt pathways by regulating CTNNB1 subcellular localization and titrating Notch activity through its ligand.

Our studies show that ROBO1 signaling is a central agent within a pathway that controls alveologenesis. We provide mechanistic insight into how ROBO1 in the basal compartment controls Notch activation in the luminal compartment by regulating the expression of JAG1 via CTNNB1. Because Notch signaling regulates the recruitment and differentiation of lineage-restricted AVPs into milk-producing alveolar cells, our model offers an explanation for why women experience a differential ability to build a milk supply during pregnancy. We further demonstrate that controlling the activation of Notch through ROBO1 has the potential to mitigate lactation insufficiency by providing a non-hormonal way to target milk production.

## MATERIALS AND METHODS

### Experimental models and subject details

#### Mouse strains

*Robo1^tm1Matl/tm1Matl^* has been previously described (Long et al., 2004); *FVB/NJ* and *C57BL/6-Tg(CAG-EGFP)1Osb/J* mice were obtained from The Jackson Laboratory and *CAnN.Cg-Foxn1^nu^/Crl* were obtained from Charles Rivers. Genotyping was performed by extracting DNA from ear-snips and performing an end-point PCR for the given transgene using the primers listed in Table S1. All animal procedures were both approved by and conducted in accordance with the guidelines set by the University of California, Santa Cruz (UCSC) Institutional Animal Care and Use Committee (IACUC).

#### Cell cultures

All cell lines were obtained from American Type Culture Collection (ATCC) and routinely checked for mycoplasma (Mycoplasma PCR kit, ABM, G238).

#### Animal studies

Nulliparous analysis was performed using adult (10-12-week-old) female mice. For timed pregnancies, adult females were checked for their estrus state as previously described (Byers et al., 2012), scored by the presence of a vaginal plug. Plugged mice were considered to be 0.5 DP on the day of the observed plug and the embryos were examined at the time of harvesting the mammary glands to confirm pregnancy state. All +/+ and –/– females used in this study were littermates or age-matched.

### Milk proxy studies

To control for olfactory bulb defects that may lead to pup rejection as previously reported in *Robo1−/−* animals (Fouquet et al., 2007), *Robo1−/−* dams were mated with *Robo1+/+* males and *Robo1+/+* dams were mated with *Robo1−/−* males to generate heterozygote pups. In addition, each litter was controlled to five pups, and their weight was recorded every 24 h post birth for 8 days.

### Mammary fat pad clearing, and transplantation

A small mammary gland tissue fragment from 8-week-old +/+ and −/− littermates was contralaterally transplanted into pre-cleared fat pads of *Foxn1^nu^* (Young, 2000) host mice. Contralateral outgrowths were harvested 17.5 DP.

### Fat pad filling analysis

Paraffin embedded +/+ and *−/−* mammary glands or contralateral outgrowths were sectioned and subjected to Hematoxylin and Eosin (H&E) staining. Images were analyzed using ImageJ, and percentage fat pad filling was calculated by measuring area occupied by the alveoli.

### Carmine Alum staining

Harvested mammary glands were spread out on a slide and allowed to dry for 1 h at room temperature. Slides were fixed overnight in Carnoy's fixative (six parts 100% ethanol, three parts chloroform, one part glacial acetic acid) at room temperature. On day 2 slides were rehydrated by incubating them in 70% ethanol twice for 10 min each, twice in 50% ethanol for 10 min each, twice in 30% ethanol for 10 min each, twice in 10% ethanol for 10 min each and in distilled water for 10 min. Slides were then stained for 2 days in Carmine Alum at room temperature. After staining, the glands were washed in 70% ethanol three times for 30 min each wash. The glands were then dehydrated by incubating them in fresh 70% ethanol once for 30 min each, twice in 95% ethanol for 30 min each and twice in 100% ethanol for 10 min each. The glands were defatted by incubating them in toluene for 2-3 days, changing the toluene solution daily before mounting using Permount (Fisher Chemical; SP15). Slides were allowed to dry before imaging them on an Axio imager scope (Zeiss).

### EdU labeling

Pregnant dams were injected intraperitoneally with EdU (50 mg/kg body weight) 18 h before harvesting. Tissue was cryosectioned at a thickness of 10 µm and subjected to immunofluorescence as described below. EdU was detected via a click-iT chemistry reaction containing the following reagents per 1 ml final reaction: 950 µl (100 mM) Tris (pH 7.5), 40 µl (100 mM) CuSO_4_, 10 µl (200 mg/ml) sodium ascorbate (C_6_H_7_NaO_6_), and 1 µl Azide-555.

### 3D cell cultures

Primary cell organoids were grown as previously described (Rubio et al., 2020). Briefly, primary cells were mixed and grown in Matrigel Growth Factor Reduced (GFR), Phenol Red-Free (Corning, CB-40230C) and cultured in basal medium. After 5 days, growing organoids were carefully washed and cultured in differentiation medium for an additional 5 days. Differentiated organoids were either imaged as 3D, or fixed and processed as previously described (Harburg et al., 2014).

### 2D cell cultures

MDA-MB-231 cells were cultured in DMEM growth medium (Gibco, 11039-021) supplemented with 10% heat-inactivated fetal bovine serum (FBS) (Corning, MT35010CV) and 1× Anti-Anti (Thermo Fisher Scientific, 15240112) at 37°C with 5% CO_2_. Undifferentiated HC11 cells were cultured in growing medium (RPMI-1640; Thermo Fisher Scientific, 72400047), supplemented with 10% FBS, 5 µg/ml insulin (Millipore-Sigma, I6634), 10 ng/ml epidermal growth factor (EGF; Preprotech, AF-100-15), 1× Anti-Anti at 37°C with 5% CO_2_. Competent HC11 cells were primed for differentiation by culturing them in priming medium [RPMI-1640 supplemented with 5% charcoal-stripped-FBS (Equitech Bio, SFBM31), 5 µg/ml insulin, 1 µM dexamethasone (Millipore-Sigma, D4902-1G) and 1× Anti-Anti] for 18 h at 37°C with 5% CO_2_. To induce differentiation, primed HC11 cells were cultured in DIP Medium [RPMI-1640, supplemented with 10% FBS, 5 µg/ml insulin, 1 µM dexamethasone, 1× anti-anti and 3 µg/ml Prolactin (NHPP, oPRL-21)] at 37°C with 5% CO_2_. Primary cells were harvested from 8- to 12-week-old mice and cultured as previously described (Macias et al., 2011; Rubio et al., 2020).

### Immunofluorescence and microscopy

Primary cells were cultured in Millicell EX 8-well slides (Millipore-Sigma, PEZGS0816) as previously described (Macias et al., 2011) and fixed with 4% formaldehyde (PFA). Cells were permeabilized during 20 min in PBS (Thermo Fisher Scientific, 10010023)+0.3% Triton X-100. Blocking of nonspecific sites was then carried out using 10% normal donkey serum (NDS) (Sigma-Aldrich, D9663) + 0.1% Triton X-100 for 60 min at room temperature. Cells were then incubated overnight at 4°C with the primary antibody diluted at the appropriate concentration in PBS +5% NDS and 0.1% Triton X-100 (see Table S3 for antibody information and dilutions). Cells were then washed three times for 15 min with PBS+0.1% Triton X-100. Secondary antibodies were added for 1 h at room temperature diluted at 1:500 in PBS +5% NDS and 0.1% Triton X-100. Cells were washed with PBS+0.1% Triton X-100 three times for 15 min. Finally, cells were washed with PBS once for 5 min before adding Vectashield^®^ Vibrance™ Mounting Media with DAPI.

Paraffin-embedded tissue was sectioned at a thickness of 5 µm and mounted on Superfrost Plus Microscope Slides (Fisher, 12-550-15). Mounted tissue was deparaffinized by warming slides to 55°C for 5 min. Slides were hydrated then by soaking in Xylene three times for 5 min, 100% ethanol twice for 2 min, 95% ethanol for 1 min, 70% ethanol for 1 min, 50% ethanol for 1 min, and diH_2_O for 10 min. Antigens were unmasked using antigen retrieval solution (VectorLabs, H3300-250) in a conventional lab microwave. Cells were permeabilized during 20 min in PBS (Thermo Fisher Scientific, 10010023)+0.1% Triton X-100. Blocking of nonspecific sites was then carried out using 10% NDS and 0.1% Triton X-100 for 60 min at room temperature in a humidifying chamber (VWR, 68432A). For antibodies raised in mouse a M.O.M.^®^ kit (VectorLabs) was used. Primary and secondary antibodies were diluted and used as described in Table S3.

Cryosections were fixed in 4% PFA for 15 min then washed with PBS three times for 5 min. Tissue was permeabilized by soaking for 15 min in PBS (Thermo Fisher Scientific, 10010023)+0.1% Triton X-100. Blocking of nonspecific sites was carried out using 10% NDS and 0.1% Triton X-100 for 60 min at room temperature. Antigen retrieval was performed using antigen retrieval solution in a conventional lab microwave. For ROBO1 staining, antigen retrieval was performed by bringing slides to a boil using 10 mM citrate-buffer (pH 6) in a conventional lab microwave and then steaming slides (Oster) for 30 min. Blocking of nonspecific sites and antibody incubations were used as described above. Samples were imaged using either a Zeiss LSM-880 confocal microscope with Airy scan or Zeiss Axiozoom Microscope as indicated.

### 3D organoids

Organoids were processed for high-resolution imaging as previously described (Rubio et al., 2020). Briefly, organoids were liberated from the 3D matrix using ice-cold recovery solution and incubating them at 4°C for 60 min. Liberated organoids were fixed with 4% PFA at 4°C for 45 min. Fixed organoids were immunostained using primary antibodies at 4°C for 18 h, washed, then incubated with secondary antibodies at 4°C for 18 h (see Table S3 for antibody information and dilutions). Immunostained organoids were mounted with Vectashield^®^ Vibrance™ Mounting Media with DAPI inside three stacked Secure-Seal™ Spacers (Thermo Fisher Scientific, S24735).

### Mammary gland CUBIC clearing

Glands were harvested from adult mice and placed in 4% PFA overnight at 4°C, then washed with PBS three times for 5 min. Fixed glands were manually cut into ∼1 cm thick pieces and processed as previously described (Matsumoto et al., 2019). Briefly, delipidation was achieved by incubating the mammary gland sections in 50% CUBIC-L (TCI, T3781) for 6 h then switching to 100% CUBIC-L for 3 days, changing it daily. Cleared tissue was washed with PBS three times for 1 h each wash, followed by immunofluorescence antibody labeling. Primary antibodies were incubated for 2 days at room temperature then washed with PBS three times for 1 h each wash. Secondary antibodies were incubated for 1 day at room temperature then washed with PBS three times for 1 h each wash (see Table S3 for antibody information and dilutions). For refracting-index matching, the stained mammary glands were incubated in 50% CUBIC-R+ (TCI, T3741) for 6 h then switched to 100% CUBIC-R+ until tissue was transparent.

### Isolation of mammary epithelial cells and flow cytometry

Mechanically dissociated inguinal, abdominal and thoracic mammary fat pads were prepared into cell suspension for FACS as previously described (Asselin-Labat et al., 2011; Shackleton et al., 2006; Shore et al., 2012; Zeng and Nusse, 2010). Briefly, mammary epithelial single cells were resuspended in HBSS supplemented with 10 mM HEPES (Gibco, 15630080) and 2% FBS. Nonspecific sites were blocked using Mouse BD Fc Block™ (BD Biosciences) for 10 min. This cell suspension was depleted of lineage-positive cells (CD45, Ter119, CD31 and BP-1) using the EasySep Mouse Epithelial Cell Enrichment Kit II as per protocol (Stem Cell Technologies, 19868) and antibody selection was performed. Mammary epithelial cells were subsequently resuspended at a density of 1×10^7^ cells/ml and stained with a combination of the following antibodies: anti-CD24 PE (Stem Cell Technologies, 60099PE.1), anti-CD29 PE-Cy7 (BioLegend, 102222), anti-CD14 FITC (eBiosiences, 11-0141-81), anti-CD45-APC (BioLegend,105826), Ter119-APC (BD Biosciences, 561033), CD31-ACP (BD Biosciences, 551262) and anti-CD117(ckit) APC-Cy7 (BioLegend, 105826) (see Table S3 for antibody information and dilutions). Cells were sorted using a BD FACS Aria II Cell Sorter and populations were analyzed using Flowjo (BD Biosciences).

### Intracellular flow cytometry

HC11 cells were detached with TrypLE™ Express Enzyme (Thermo Fisher Scientific, 12604013), washed with cold 1× PBS and passed through a 45 µm filter. Single cells were fixed with 1.5 ml of ice-cold methanol at −20°C for 10 min. Fixed cells were centrifuged and washed with 1× PBS supplemented with 1% bovine serum albumin (BSA). Nonspecific sites were blocked using Mouse BD Fc Block™ for 10 min. Blocked cells were incubated with primary antibody for 1 h at room temperature, washed, then incubated with secondary antibody for 1 h at room temperature. Cells were sorted and analyzed as described above.

### Chromatin immunoprecipitation (ChIP) analysis

ChIP was performed using a Cut&Run kit (Cell Signaling Technology) as per the manufacturer's protocol. Briefly, 100,000 FACS-purified basal cells (Lin−; CD49f^hi^; CD24+) were used per condition. Each prep was incubated with either ChIP-validated rabbit anti-CTNNB1 monoclonal antibody (active-β-catenin, Clone D13A1) or rabbit IgG Isotype control (clone DA1E) overnight at 4°C. ChIP DNA was recovered using phenol/chloroform extraction followed by ethanol precipitation as per the manufacturer's protocol. Quantification of DNA by qPCR was performed using equal amounts of DNA. qPCR analysis was performed in triplicates using SimpleChIP^®^ Universal qPCR Master Mix (Cell Signaling Technology, 88989). The reactions were run in a Bio-Rad CFX'Connect Real-Time System and CFX Manager software (Bio-Rad) as follows: 95°C for 3 min followed by 40 cycles of 95°C for 15 s, 60°C for 60 s. Sample normalization was performed using the signal from the Sample Normalization Spike-in yeast DNA with a primer set specific to the *Saccharomyces cerevisiae ACT1* gene, provided in the Cut&Run kit.

### *In vivo* GSI

GSI RO4929097 (MedchemExpress, HY-11102) was orally administered at 10 mg/kg body weight for 5 days as previously described (Regan et al., 2013). Mammary glands were harvested after 5 days of GSI or vehicle treatment and prepared for single cell analysis. For *in vitro* studies, HC11 cells were treated with 10 nM for 36-48 h before harvest or differentiation for lactogenic studies.

### Western blotting

Whole cell lysates were prepared using 1× NP40 lysis buffer (Thermo Fisher Scientific, FNN0021) supplemented with Pierce Protease and Phosphatase inhibitors (Thermo Fisher Scientific, A32959). Cells were washed with ice-cold PBS (Gibco, 14190136) and lysed direct in buffer and kept at 4°C rocking at 70 rpm. Lysed cells were collected and then spun at 14,000 ***g*** at 4°C for 15 min. Subcellular fractionations were prepared using a ProteoExtract^®^ Subcellular Proteome Extraction Kit as per manufacturer's protocol (Millipore-Sigma, 539790). Equivalent (35-50 μg) samples were resolved by SDS page and transferred to polyvinylidene difluoride (PVDF) (Millipore-Sigma, IPVH00010) for 60 min at 100 V. Immunoblots were blocked using either 5% non-fat milk, 5% BSA or 5% (%v/v) fish gelatin for 60 min at room temperature. Primary antibodies were incubated overnight at 4°C in a rocker at 65 rpm All HRP-conjugated secondary antibodies (The Jackson Laboratory) were used at 1:7500 for 90 min at room temperature (see Table S3 for antibody information and dilutions). Immunoblots were developed using Clarity ECL (Bio-Rad), detected using a Bio-Rad Chemi-Doc MP Image, and quantified using ImageLab software (Bio-Rad) as previously described (Le et al., 2016).

### RNA preps and RT-qPCR

Total RNA was harvested from FACS-purified cells lysed in TRIzol reagent (Invitrogen) and phase separated according to the manufacturer's protocol with an additional overnight RNA precipitation step in ethanol (Macias et al., 2011). Whole-gland total RNA was extracted using Direct-zol RNA MiniPrep Plus (Zymo, R2070).The RNA was further purified with TURBO DNase (Ambion, AM1906) treatment. Total RNA quality was analyzed by agarose gel electrophoresis and quantified using an ND-1000 spectrophotometer (NanoDrop). cDNA was prepared from 500-1000 ng of total RNA using iScript cDNA synthesis kit (Bio-Rad, 1708841). Quantitative RT-qPCR was performed in triplicates using SsoAdvanced Universal SYBR Green Supermix, (Bio-Rad, 1725272). The reactions were run in a Bio-Rad CFX'Connect Real-Time System and CFX Manager software (Bio-Rad) as follows: 95°C for 2 min followed by 40 cycles of 95°C for 15 s, 60°C for 30 s and 72°C for 45 s. The melting curve was graphically analyzed to control for nonspecific amplification reactions. Results were normalized to GAPDH. See Table S2 for primer sequences.

### RNA-seq library preparation

Total RNAs were then extracted from FACS-purified luminal progenitor cells, harvested from *Robo1*+/+ or *Robo1*−/− mice (*n*=3 per genotype, two animals per *n*) using TRIreagent LS (Sigma-Aldrich, T3934). Poly(A)+ RNA sequencing libraries were made from each sample using the TruSeq RNA library preparation kit v.1 (Illumina). A total of six libraries were created by PCR amplification with Illumina barcoding primers using kit recommended conditions and quantified using a Bioanalyzer DNA 1000 kit (Agilent). Libraries were then pooled in equimolar amounts and sequenced on a HiSeq 2000 Sequencing system (Illumina). All high-quality reads were trimmed to 75×75 bp using custom scripts. We used bowtie2 to map reads to mouse repeat elements (Langmead and Salzberg, 2012). Repeat filtered reads were then mapped to the mouse genome (assembly NCBI37/mm9) using TopHat (Kim et al., 2013). DESeq2 was used to calculate normalized read counts within genes, calculate fold change in gene expression and estimate *P*-values and adjusted *P*-values for change in gene expression values (Love et al., 2014).

### Image processing

Images were processed using Fiji or ZEISS ZEN Imaging Software (Zeiss) and equally adjusted manually if needed. All graphs were generated with Graphpad Prism version 9.0.

### Quantification and statistical analysis

Sample size and statistical significance between conditions is denoted in the figure legends. For multiple group comparison, a one-way ANOVA analysis was performed followed by a two-tailed Student's *t*-test (unpaired or paired as described in the figure legends). We performed similar analyses with different tests (e.g. a paired *t*-test or a Student's *t*-test on the un-normalized data) yielding *P*-values of comparable significance. For the milk proxy analysis, a two-way ANOVA was performed followed by a two-tailed, unpaired Student's *t*-test. All error bars represent s.e.m., and significance is denoted in each figure bar. *P*-values higher than 0.05 were considered not statistically significant.

## Supplementary Material

Supplementary information

Reviewer comments
