# Supplementary material for: Alveolar progenitor differentiation and lactation depends on paracrine inhibition of notch via ROBO1/CTNNB1/JAG1
Source: Development. 2021 Nov 11;148(21):dev199940. doi: 10.1242/dev.199940 (PMC8627605; doi:10.1242/dev.199940)
Supplement: Supplementary information [file develop-148-199940-s1.pdf]

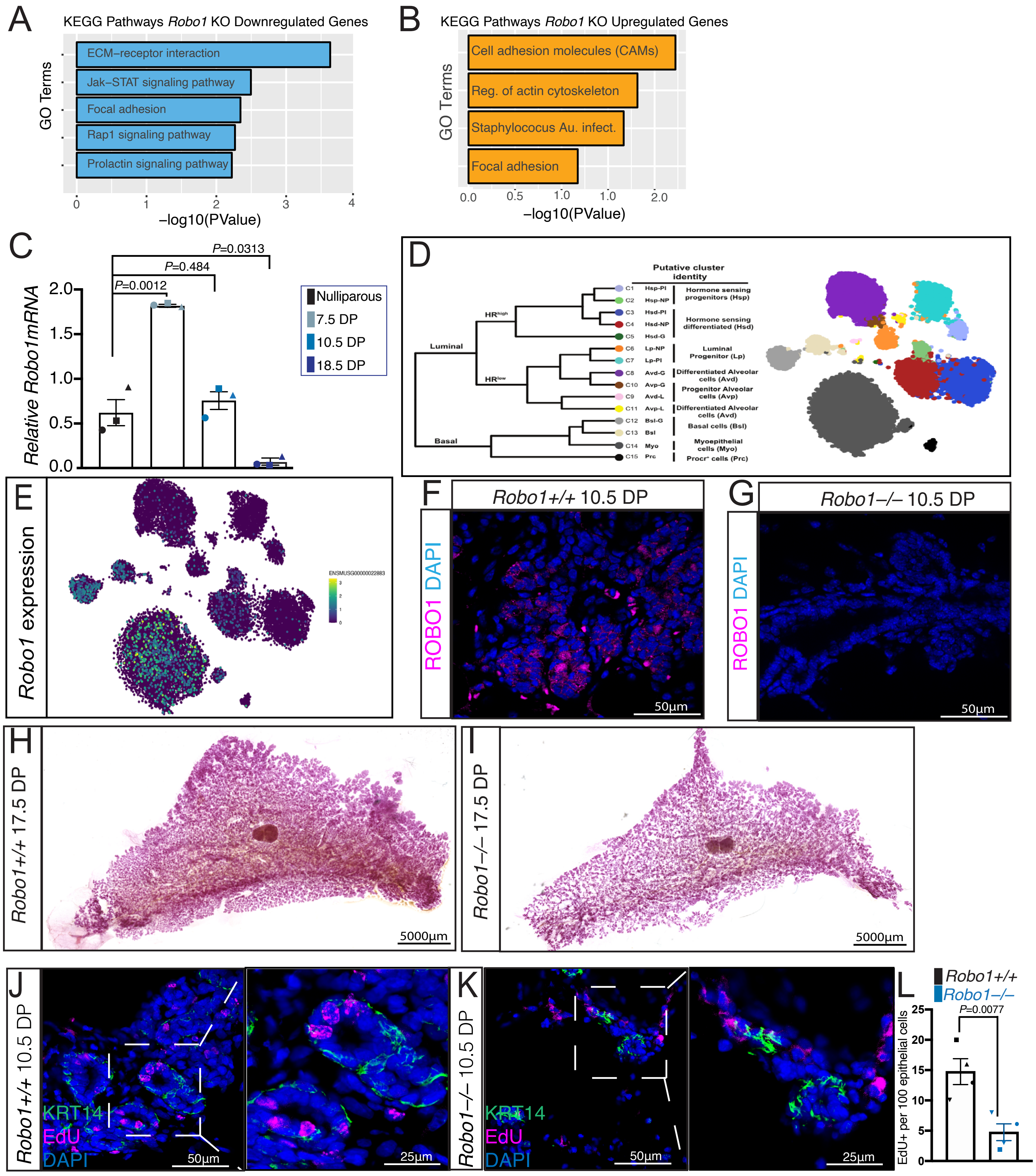

Fig. S1. (related to Figure 1): (A, B) KEGG pathway enrichment analysis for *Robo1-/-* LPs down- (A) and up- (B) regulated genes. (C) RT-qPCR for *Robo1* in whole MGs shows peak level at 7.5DP relative to nulliparous. (D, E) Dendogram (D) of mammary epithelial cell clusters and tSNE plots (E) show *Robo1* expression as determined by single-cell RNA-Seq data from Bach and colleagues (Bach et al., 2017). (F, G) Representative confocal images show luminal ROBO1 (magenta) in sections of *Robo1*+/+ 10.5DP alveoli (F) and no staining in *Robo1-/-* 10.5DP alveoli (G). (H, I) Representative carmine-stained 17.5DP whole mounts of *Robo1*+/+ (H) and *Robo1-/-* (I) littermates. (J- L) Representative confocal images of EdU labeling (magenta) and Keratin 14 (KRT14) (green) (J, K) and quantification (L) of mammary epithelial cells show reduced proliferation in 10.5DP *Robo1-/-* MG tissue (K, L). n=3 independent experiments except L n=4, 3 images/n. Statistical analysis was performed using an unpaired Student *t*-test with Welch's correction. Data are represented as mean ± SEM.

Development • Supplementary information

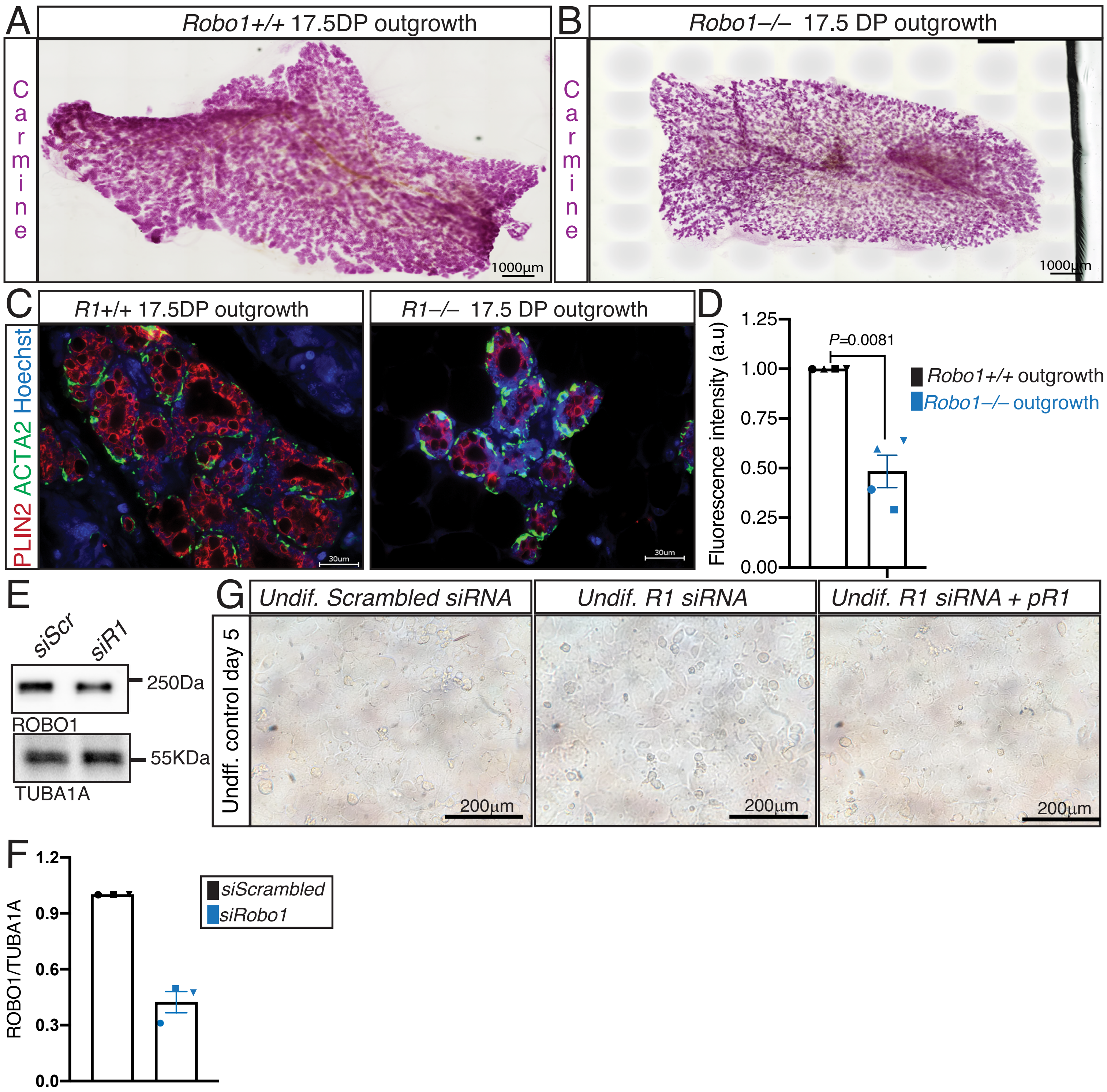

Fig. S2. (related to Figure 2): (A, B) Representative carmine-stained whole mounts of 17.5DP *Robo1+/+* (A) and *Robo1-/-* (B) outgrowths. (C, D) Representative images (C) and quantification (D) shows reduced PLIN2 (red) with ACTA2 (green) in 17.5DP *Robo1-/-* outgrowth sections. (E, F) Immunoblot and quantification show *Robo1* KD using siRNA (two-tailed paired *t*-test). (G) DIC images show no milk dome formation after control (*Scramble*) or *Robo1* siRNA transfection into undifferentiated HC11 cells even after *Robo1* overexpression. n=3 independent experiments except D n=4, 3 images/n for D. Statistical analysis was performed using an unpaired Student *t*-test with Welch's correction or as stated above. Data are represented as mean ± SEM.

Development • Supplementary information

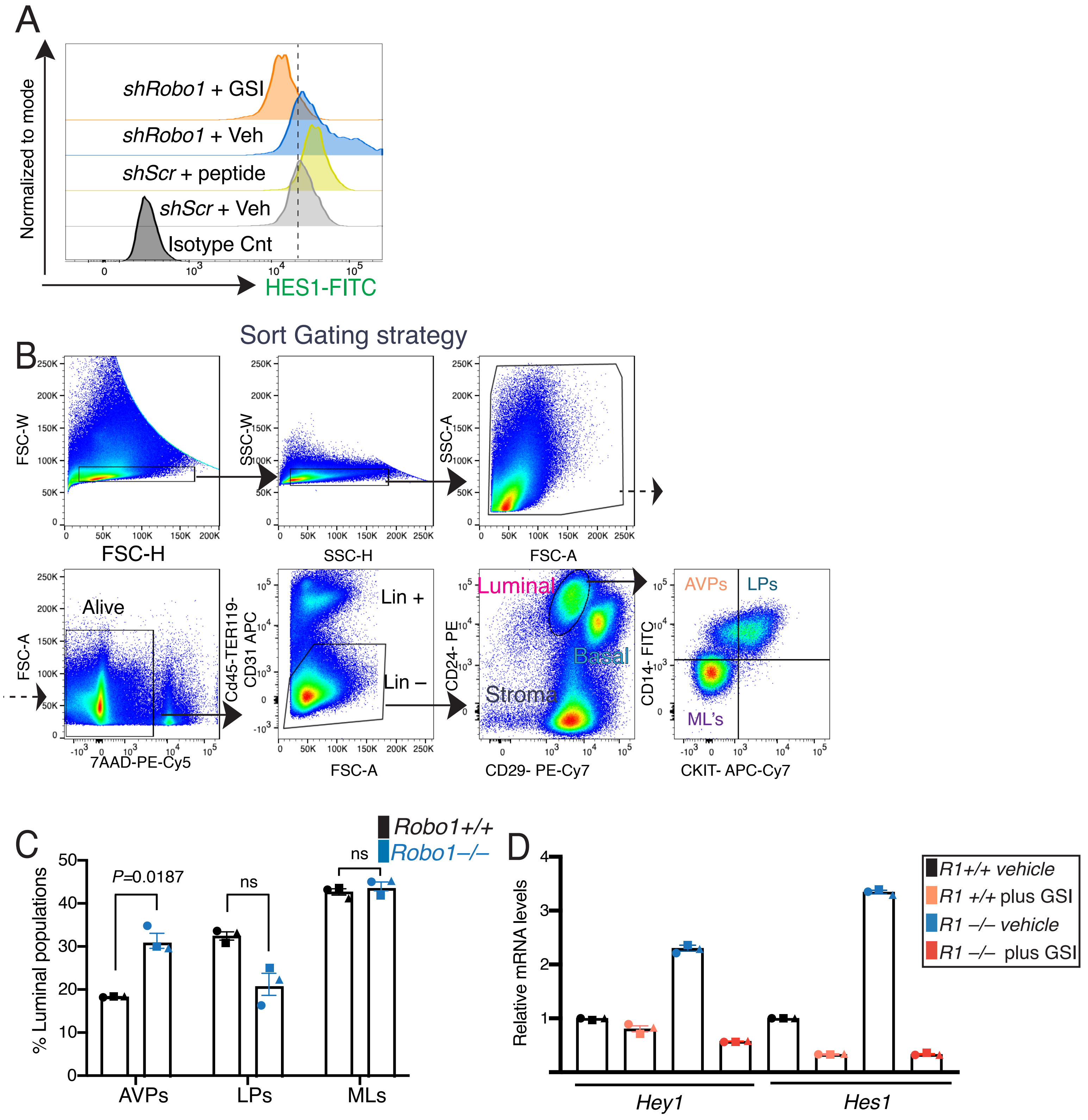

Fig. S3. (related to Figure 3): (A) Histogram of intracellular HES1 staining via FACS shows increased HES1 signal with JAG1 peptide, and also with *Robo1* KD that is prevented by GSI. (B) Flow cytometry gating strategy. (C) FACS quantification of *Robo1*+/+ and *Robo1*-/- luminal subpopulations. (D) Validation of GSI treatment through RT-qPCR of Notch effectors from vehicle and GSI-treated, FACS-sorted AVPs. All experiments: n=3 independent experiments except D is n=1 and shows technical replicates. Statistical analysis was performed using a one-way ANOVA followed by an unpaired Student *t*-test with Welch's correction. Data are represented as mean ± SEM.

Development • Supplementary information

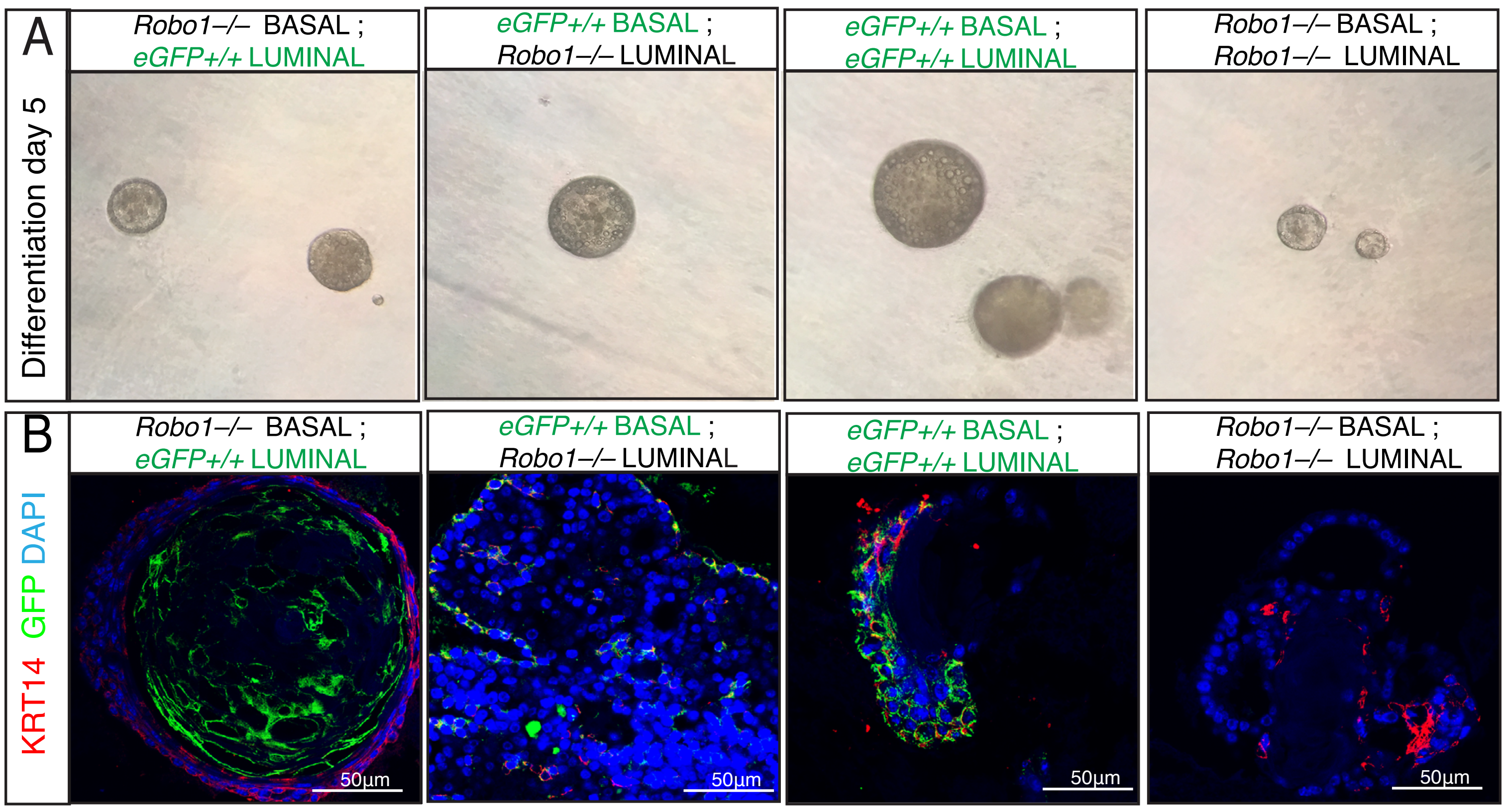

Fig. S4. (related to Figure 4): (A) DIC images of differentiated (day 5) organoids cultured in Matrigel show larger *eGFP+/+/eGFP+/+* organoids, smaller *Robo1-/-/Robo1-/-* organoids and intermediate sized mosaic organoids: *Robo1-/-/eGFP+/+, eGFP+/+/Robo1-/-*. (B) Representative confocal images of paraffin-embedded organoid sections show GFP (green) and basal marker KRT14 (red).

Development • Supplementary information

Figure S5, Related to Figure 5

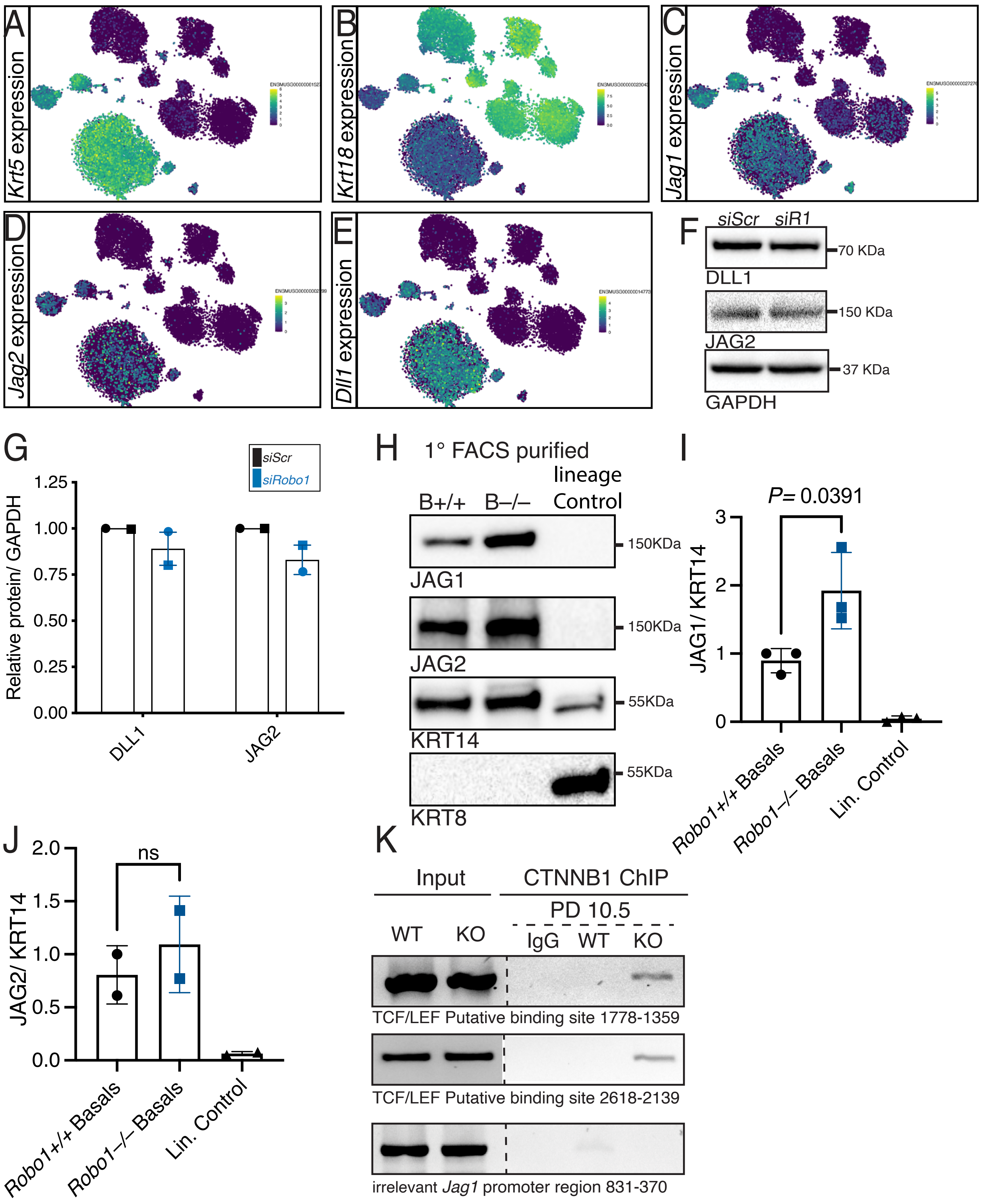

Fig. S5. (related to Figure 5): (A-E) tSNE plots showing MG epithelial cells markers basal KRT5 (A) and KRT18 (B) and Notch ligand expression (C-E) as determined by single-cell RNA-Seq data (Bach et al., 2017). (F, G) Immunoblot (F) and quantification (G) show no change in JAG2 or DLL1 expression with *Robo1* KD in HC11 cells. (H-J) Immunoblot (H) and quantification (I, J) show increased JAG1, but not a significant increase of JAG2, in *Robo1*-/- FACS-purified basal cells (two-tailed paired *t*-test). (K) PCR fragments, amplified using primers within the *Jag1* promoter either specific to a Tcf/Lef binding site or to an irrelevant location (control), show that chromatin immunoprecipitation with anti-CTNNB1 is enriched in DP10.5 basal *Robo1*-/- cells. n=3 independent experiments except L is n=3. Data are represented as mean ± SEM.

Development • Supplementary information

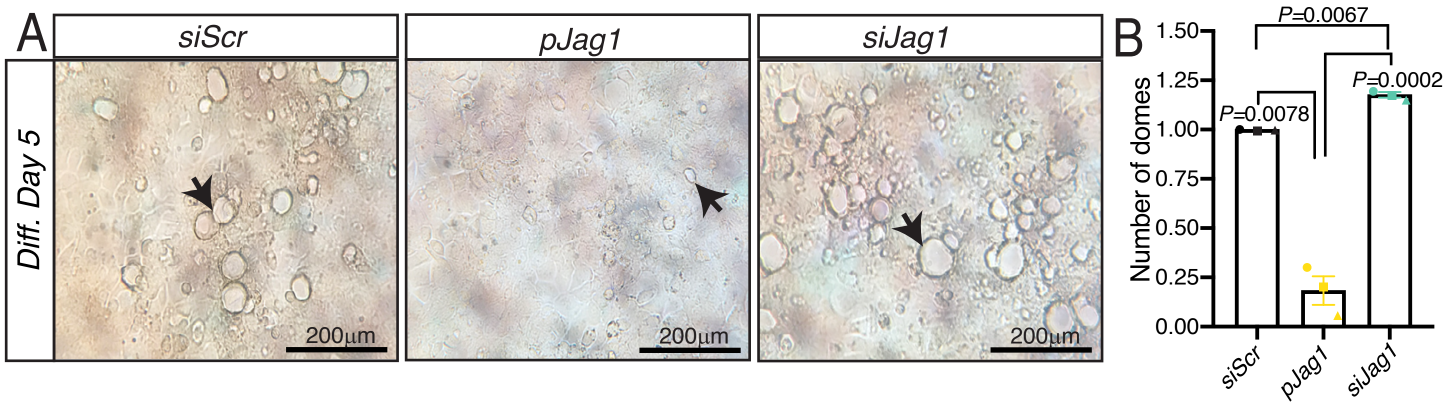

Fig. S6. (related to Figure 6): (A, B) DIC images (A) and quantification (B) of differentiated HC11 cells transfected with either *Scramble*, *pJag1* or *siJag1* to overexpress or KD *Jag1* show reduced and enhanced milk dome formation, respectively. Arrows identify domes. n=3 independent experiments. Statistical analysis was performed using a one-way ANOVA followed by an unpaired Student *t*-test with Welch's correction. Data are represented as mean ± SEM.

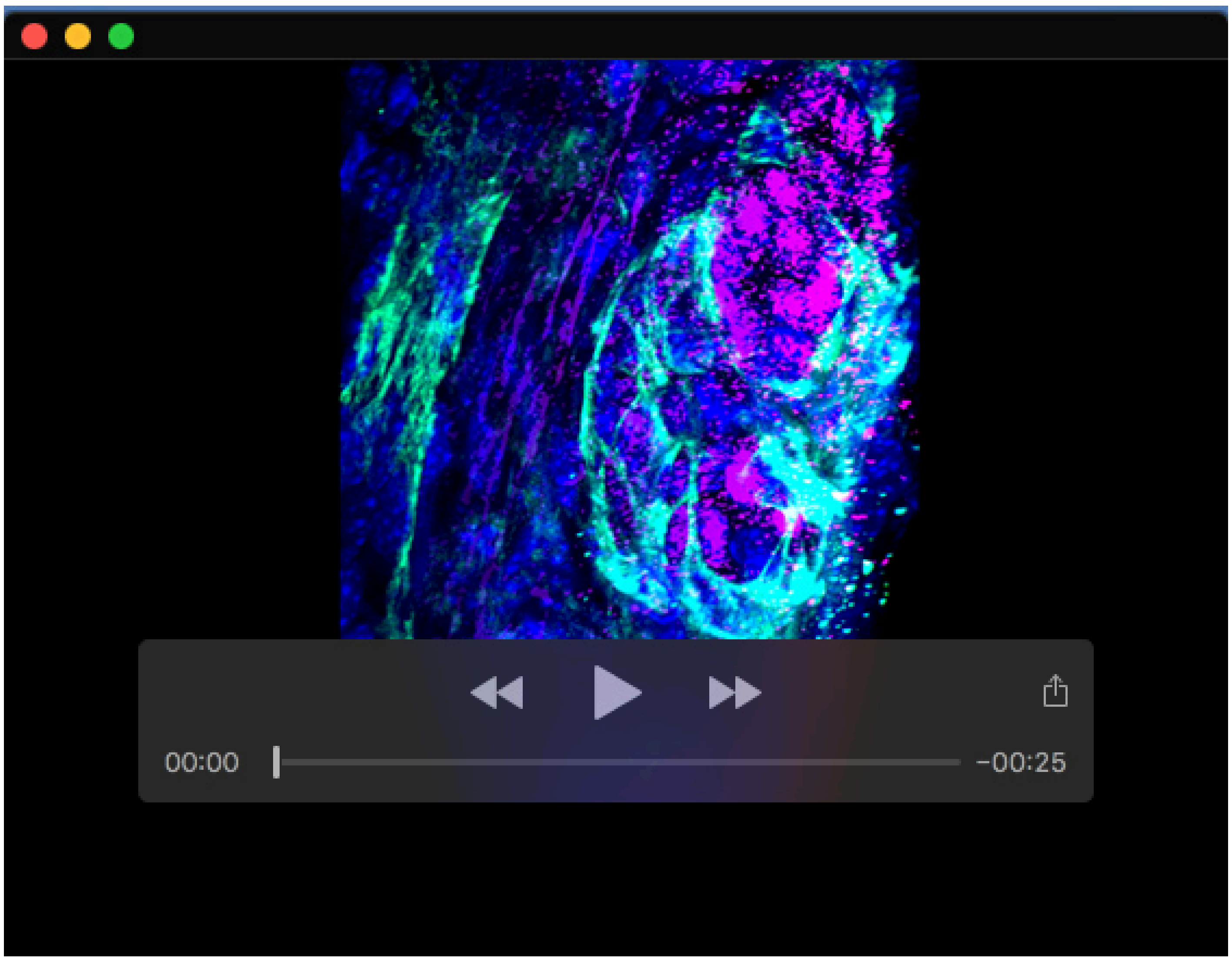

Movie 1. 3D confocal movie of CUBIC cleared alveoli from 7.5DP *Robo1*$^{+/+}$ tissue showing ROBO1 (magenta) with basal marker smooth muscle actin (ACTA2) (green), and ROBO1 (magenta) in an underlying luminal cell.

Table S1. Reagents

Click here to download Table S1

Table S2. Oligonucleotides

Click here to download Table S2

Table S3. Antibodies and dilutions

Click here to download Table S3

Development • Supplementary information
