# Supplementary material for: Alveolar progenitor differentiation and lactation depends on paracrine inhibition of notch via ROBO1/CTNNB1/JAG1
Source: Development. 2021 Nov 11;148(21):dev199940. doi: 10.1242/dev.199940 (PMC8627605; doi:10.1242/dev.199940)
Supplement: Reviewer comments [file develop-148-199940_review_history.pdf]

Alveolar progenitor differentiation and lactation depends on paracrine inhibition of notch via ROBO1/CTNNB1/JAG1

Oscar Cazares, Shamila Chatterjee, Pinky Lee, Catherine Strietzel, J. W. Bubolz, Gwyndolen Harburg, Jon Howard, Sol Katzman, Jeremy Sanford and Lindsay Hinck

DOI: 10.1242/dev.199940

Editor: Liz Robertson

Review timeline
Original submission: 28 June 2021
Editorial decision: 20 July 2021
First revision received: 13 September 2021
Accepted: 23 September 2021

Original submission

First decision letter

MS ID#: DEVELOP/2021/199940

MS TITLE: Alveolar Progenitor Differentiation and Lactation Depends on Paracrine Inhibition of Notch via ROBO1/CTNNB1/JAG1

AUTHORS: Oscar E Cazares, Shamila E Chatterjee, Pinky Lee, Catherine E Strietzel, J W Bubolz, Gwyndolen E Harburg, Jon E Howard, Sol E Katzman, Jeremy E Sanford, and Lindsay E Hinck

I have now received all the referees reports on the above manuscript, and have reached a decision. The referees' comments are appended below, or you can access them online: please go to BenchPress and click on the 'Manuscripts with Decisions' queue in the Author Area.

The overall evaluation is positive and we would like to publish a revised manuscript in Development, provided that the referees' comments can be satisfactorily addressed. Please attend to all of the reviewers' comments in your revised manuscript and detail them in your point-by-point response. If you do not agree with any of their criticisms or suggestions explain clearly why this is so.

We are aware that you may currently be unable to access the lab to undertake experimental revisions. If it would be helpful, we encourage you to contact us to discuss your revision in greater detail. Please send us a point-by-point response indicating where you are able to address concerns raised (either experimentally or by changes to the text) and where you will not be able to do so within the normal timeframe of a revision. We will then provide further guidance. Please also note that we are happy to extend revision timeframes as necessary.

Reviewer 1

Advance summary and potential significance to field

This manuscript focuses on defining the effects of ROBO1 deletion in mammary epithelial cells on alveolar development during pregnancy. The authors describe a model in which ROBO1 in the basal

© 2021. Published by The Company of Biologists under the terms of the Creative Commons Attribution License (https://creativecommons.org/licenses/by/4.0/).

epithelial cells limits expression of JAG1 as a mechanism to promote lobuloalveolar differentiation and limit the alveolar progenitor population. The results demonstrate that loss of ROBO1 in the basal epithelial cells leads to increased JAG1 expression, which is associated with enhanced Notch signaling in the luminal epithelium, reduced differentiation, and expansion of alveolar progenitors. The results and conclusions are convincing, and the experiments include appropriate controls. A variety of in vivo and in vitro models are used to convincingly demonstrate the presence of a paracrine signaling pathway between the basal and luminal epithelial compartments involving ROBO1,CTNNB1 and JAG1 that is important for proper alveolar differentiation.
Understanding these complex mechanisms of interaction is critical for understanding mammary gland development and lactation.

Comments for the author

1) Lines 113-116: The authors should clarify in the text that the RNA-seq was performed on cells from Robo1-knockout mice.
2) Lines 129-130: The authors should clarify in the text that the RT-qPCR analysis was performed on RNA isolated from whole mammary glands.
3) Line 222: The authors should consider changing their wording regarding "robust milk production" and "produced little or no milk" to phrases such as "expression of milk proteins", given that it is unclear whether these cells are actually producing milk based on CSN2 staining alone.
4) Lines 225-227: These sentences should refer to Figure 4, rather than Figures 5 and 6.
5) Line 242: Correct to "MDA-MB-231"
6) Lines 255-257: Should refer to Figure 5G.
7) Figure 5G: The authors should provide quantification of these data. There appears to be some nuclear CTNNB1 staining in a few places in the Robo1+/+ organoids- is there any difference in nuclear CTNNB1 staining in non-KRT14 positive cells?
8) Figure 6: Were expression levels of Notch effectors (such as Hes1) examined in the luminal epithelial cells to confirm alterations in Notch activity?
9) Figure 1L: The day 8 timepoint of lactation is not labeled as statistically significant- does this suggest that the mammary glands from Robo1 knockout mice recover during lactation?

<u>Reviewer 2</u>

Advance summary and potential significance to field

The present manuscript by Cazares et al. addresses the role of Robo1 in the mammary epithelium in late pregnancy. The authors reveal a role in lactogenic differentiation and identify a basal-luminal paracrine interaction mediated by jagged 1 downstream of b-catenin activation triggered by robo-1. They claim that alveolar progenitor cells fail to differentiate. The data a very clean and nicely presented. The conclusions, however, should be drawn more carefully.

Comments for the author

Major concern:
The authors very nicely demonstrate an epithelial-intrinsic role for Robo1 in the mammary gland at d17.5 of pregnancy. For a developmental defect it is important to show when the phenotype becomes apparent. Alveologenesis follows side branching during pregnancy around d14.5. Are the mutant glands undistinguishable from their wt counterparts up to this point or could they already show a proliferative block in virgin females that would confound the interpretation of the phenotype at d17.5 of pregnancy? Critical developmental milestones 3 weeks (prepuberty), 5-6 weeks (puberty), 8-10 weeks (adulthood) and earlier stages of pregnancy should be examined, if necessary in the epithelial transplant setting.
It is confusing that only the RNASeq of luminal progenitors were compared. Were there differences between mt and wt basal and mature luminal cells? What were the ratios of the different cell populations in wt and mt females?
Line 242: MDA-MB-23? Supposedly, MDA-MB-231 cells here? These are not basal mammary cells. MCF10A cells are a good model for basal mammary epithelial cells and should be used.

© 2021. Published by The Company of Biologists under the terms of the Creative Commons Attribution License (https://creativecommons.org/licenses/by/4.0/).

Line 276/277. The conclusion is an overstatement. The nice in vitro data indicate an important role for jagged1 in lactogenic differentiation as assessed by CSN2 expression. The term alveologenesis only applies to the in vivo phenomenon that physiologically occurs during pregnancy.

Minor concerns:
1. Running title (and throughout the ms): there are no lobules in the mouse mammary glands should read “mammary alveologenesis”
2. Abstract: line 46: “Breast” is used only for the human organ here: mammary gland 3.
Figure 1B is puzzling: DP7.5 (typo??) there is no alveologenesis. “In the developing alveoli” is imprecise, on a section you cannot tell small branches from alveoli. If thick section was used to ensure 3D interpretation, please mention.
4. Line 166 “2D organoids” does not make sense, better refer to ability to form domes on plastic.
5. Wnt4 was shown to activate canonical Wnt signaling in the basal epithelium as assessed by axin-2 transcription. This is important for mammary stem cell function but not alveologenesis (Rajaram RD et al EMBO 2015). How may the wnt4 induced Wnt signaling in basal cells interact with the one induced by Robo-1 here?

First revision

Author response to reviewers' comments

Reviewer 1 Advance Summary and Potential Significance to Field:
This manuscript focuses on defining the effects of ROBO1 deletion in mammary epithelial cells on alveolar development during pregnancy. The authors describe a model in which ROBO1 in the basal epithelial cells limits expression of JAG1 as a mechanism to promote lobuloalveolar differentiation and limit the alveolar progenitor population. The results demonstrate that loss of ROBO1 in the basal epithelial cells leads to increased JAG1 expression, which is associated with enhanced Notch signaling in the luminal epithelium, reduced differentiation, and expansion of alveolar progenitors. The results and conclusions are convincing, and the experiments include appropriate controls. A variety of in vivo and in vitro models are used to convincingly demonstrate the presence of a paracrine signaling pathway between the basal and luminal epithelial compartments involving ROBO1, CTNNB1 and JAG1 that is important for proper alveolar differentiation. Understanding these complex mechanisms of interaction is critical for understanding mammary gland development and lactation.

Reviewer 1 Comments for the Author:
1) Lines 113-116: The authors should clarify in the text that the RNA-seq was performed on cells from Robo1-knockout mice.

We thank the reviewer for suggesting this point of clarification. The following text was added:

Lines 113-117: *To identify cellular processes that may be regulated by ROBO1, we performed fluorescence-activated cell sorting (FACS) to purify populations of cells harvested from WT and Robo1$^{tm1Matl/tm1Matl}$ (herein referred to as Robo1-/- or KO) mature, virgin MGs: basal cells (Lin^{-} CD24^{+}CD29^{hi}; BC), mature luminal cells (Lin^{-}CD24^{lo}CD29^{+}CD61^{-}; ML), and luminal progenitor cells (Lin^{-}CD24^{lo}CD29^{+}CD61^{+}; LP) (Harburg et al., 2014), and performed RNA-seq analysis.*

2) Lines 129-130: The authors should clarify in the text that the RT-qPCR analysis was performed on RNA isolated from whole mammary glands.

Thank you for pointing out this oversight. We have corrected this as shown below in the main text; we also included this clarification in the Supplemental Figure Legend:

Lines 130-131: *To investigate a putative role for ROBO1 during pregnancy, we evaluated its gene expression in whole MGs by RT-qPCR and observed a peak in its expression at 7.5 day pregnancy*

© 2021. Published by The Company of Biologists under the terms of the Creative Commons Attribution License (https://creativecommons.org/licenses/by/4.0/).

(DP) (Fig S1C).

3) Line 222: The authors should consider changing their wording regarding “robust milk production” and “produced little or no milk” to phrases such as “expression of milk proteins”, given that it is unclear whether these cells are actually producing milk based on CSN2 staining alone.

We have clarified this (and other) descriptions to be precise as follows.

Lines 221-224: *This robust production of the CSN2 milk protein was also observed in WT/WT organoids (Fig 4D, F), whereas, KO/KO organoids were similar to KO/WT organoids and produced little or no CSN2 (Fig 4E, F).*

4) Lines 225-227: These sentences should refer to Figure 4, rather than Figures 5 and 6.

We apologize for the oversight. The correct callouts for Figure 4 have added.

5) Line 242: Correct to “MDA-MB-231”

We thank the reviewer for pointing this typo out. Based on a suggestion from Reviewer 2, we removed the data on the immortalized MDA-MB-231 cell line because the line is not an optimal model for basal cells. We replaced these data with a Western blot showing increased JAG1, but not JAG2, in FACS-purified populations of *Robo1+/+* and *Robo1-/-* basal cells harvested from virgin mice.

Line 241-242: *We also examined JAG expression in FACS-purified populations of Robo1+/+ and Robo1-/- basal cells and found more JAG1 in KO, compared to WT, cells and no significant change in JAG2 (Fig S5H-J).*

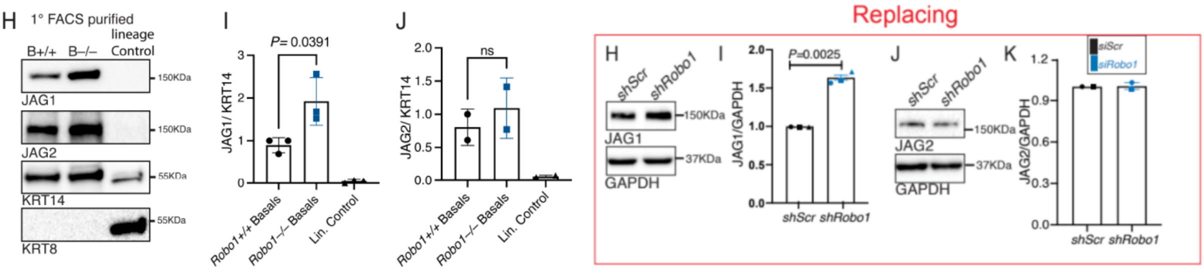

Figure 1: Left (revised Figure 5 H-J): Immunoblot (H) and quantification (I, J) show increased JAG1, but not JAG2, in *Robo1-/-* FACS-purified basal cells (two-tailed paired *t*-test). **Right (replaced Figure 5H-J)**.

6) Lines 255-257: Should refer to Figure 5G.

We apologize for the omission. The correct call-outs for Figure 5G have been added.

7) Figure 5G: The authors should provide quantification of these data. There appears to be some nuclear CTNNB1 staining in a few places in the Robo1+/+ organoids- is there any difference in nuclear CTNNB1 staining in non-KRT14 positive cells?

In the response to the Reviewer’s request, we quantified the data and include the following description of it in the revised manuscript.

Lines 255-256: *We observed more nuclear expression of CTNNB1 in Robo1-/- basal cells and no significant change in luminal cells (Fig. 5H).*

© 2021. Published by The Company of Biologists under the terms of the Creative Commons Attribution License (https://creativecommons.org/licenses/by/4.0/).

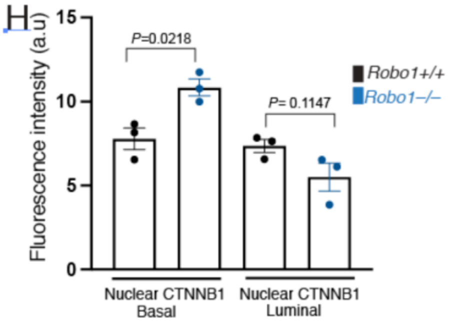

Figure 2 (revised manuscript, Figure 5H): Quantification (H) of differentiated *Robo1*+/+ and *Robo1*–/– organoids show increased nuclear CTNNB1 (green) staining in *Robo1*–/– KRT14+ (magenta) basal cells. n=3 independent experiments.

8) Figure 6: Were expression levels of Notch effectors (such as Hes1) examined in the luminal epithelial cells to confirm alterations in Notch activity?

Thank you for the suggestion. We performed HES1 staining on the organoids and include the data in revised Figure 6. As we observed for JAG1, (lines 274-279) *we observed little HES1 expression in the basal cells of Robo1+/+ organoids infected with control shScr (Fig. 6A-C). As expected, these organoids displayed lumenal CSN2 accumulation, indicating robust differentiation (Fig. 6D, E). In contrast, JAG1 and nuclear HES1 expression were upregulated in Robo1-/- organoids infected with control shScr (Fig. 6A-C), and there was no detectable CSN2 immunostaining (Fig. 6D, E), indicating that the luminal cells of these organoids did not differentiate into milk-producing alveolar cells. However, KD of Jag1 reduced both JAG1 and HES1 expression (Fig. 6A-C), and rescued the differentiation of Robo1-/- organoids as shown by CSN2 expression (Fig. 6D, E).*

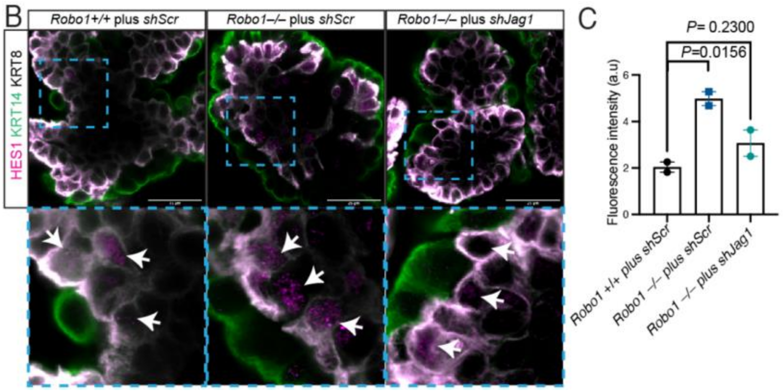

Figure 3 (revised Figure 6B, C): (**B, C**) Representative 3D confocal images (B) and quantification (C) of *Robo1*+/+ and *Robo1*-/- organoids infected with either *shScr* or *shJag1* and stained for HES1 (magenta) and KRT14 (green) show increased HES1 expression in *Robo1*-/- basal cells that is decreased by KD of *Jag1* (*shJag1*).

9) Figure 1L: The day 8 timepoint of lactation is not labeled as statistically significant- does this suggest that the mammary glands from Robo1 knockout mice recover during lactation?

We thank the reviewer for pointing this out. We see now that the quantification was dropped from the manuscript when we rendered the figure into the pdf format. We include the p-value at day 8 in the revised manuscript (p = 0.0176). To answer your question, since the developing pups can begin eating solid chow starting at day 11, we decided to show the data up until day 8 when we can comfortably say that the measured pup weight is dependent on the mother’s milk supply. Below we show the p-values and graph out to day 12, when the pups are eating chow. Heterozygous pups fed by *KO* dams still weigh less than heterozygous pups fed by *WT* dams up to this day.

© 2021. Published by The Company of Biologists under the terms of the Creative Commons Attribution License (https://creativecommons.org/licenses/by/4.0/).

Day	P value
0	0.3739
1	0.01442
2	0.01344
3	0.00144
4	0.01442
5	0.00233
6	0.00393
7	0.00324
8	0.01755
9	0.01121
10	0.04206
11	0.02839
12	0.00733

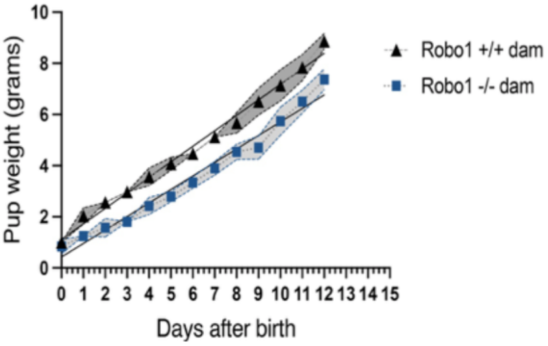

Figure 4: Quantification out to day 12 shows pups fed by *Robo1-/-* dam gain less weight (L) (two-way ANOVA followed by a two-tailed *t*-test). In the revised manuscript we show quantification out to day 8 with inclusion of the previously omitted p-value at day 8.

Reviewer 2 Advance Summary and Potential Significance to Field:
The present manuscript by Cazares et al. addresses the role of Robo1 in the mammary epithelium in late pregnancy. The authors reveal a role in lactogenic differentiation and identify a basal-luminal paracrine interaction mediated by jagged 1 downstream of b-catenin activation triggered by robo-1. They claim that alveolar progenitor cells fail to differentiate. The data a very clean and nicely presented. The conclusions, however, should be drawn more carefully.

Reviewer 2 Comments for the Author:

Major concern:

The authors very nicely demonstrate an epithelial-intrinsic role for Robo1 in the mammary gland at d17.5 of pregnancy. For a developmental defect it is important to show when the phenotype becomes apparent. Alveologenesis follows side branching during pregnancy around d14.5. Are the mutant glands undistinguishable from their wt counterparts up to this point or could they already show a proliferative block in virgin females that would confound the interpretation of the phenotype at d17.5 of pregnancy? Critical developmental milestones 3 weeks (prepuberty), 5-6 weeks (puberty), 8-10 weeks (adulthood) and earlier stages of pregnancy should be examined, if necessary, in the epithelial transplant setting.

We have previously documented the *Robo1-/-* phenotype in virgin mammary glands (Macias et al., 2011). Interestingly, at this stage we identified exuberant branching in the *Robo1* KO that we tracked to excess proliferation of the basal cells (Figure 5A-C). Thus, lack of proliferation at an earlier stage of development does not explain the lack of alveologenesis observed during pregnancy. Indeed, the *Robo1-/-* gland appears primed for robust alveolar development upon pregnancy, but we found in the current manuscript that this does not occur. Instead, we showed by EdU labeling reduced proliferation of epithelial cells in *Robo1-/-* mammary gland tissue at pregnancy day 10.5 (manuscript Fig. S1J-L). Here, we supply whole mount images of *WT* and *Robo1 -/-* mammary glands at pregnancy day 14.5. We see that the *WT* mammary gland is more developed with larger nascent alveolar structures compared to the *KO* (Figure 5D). In the current manuscript, we show H&E analysis at a later timepoint (pregnancy day 17.5) that reveals, in the *Robo1-/-* mammary gland tissue, immature alveolar structures, which have failed to fully differentiate into milk producing alveoli (Figure 5E and manuscript Fig. 1E).

It is confusing that only the RNASeq of luminal progenitors were compared. Were there differences between mt and wt basal and mature luminal cells? What were the ratios of the different cell populations in wt and mt females?

© 2021. Published by The Company of Biologists under the terms of the Creative Commons Attribution License (https://creativecommons.org/licenses/by/4.0/).

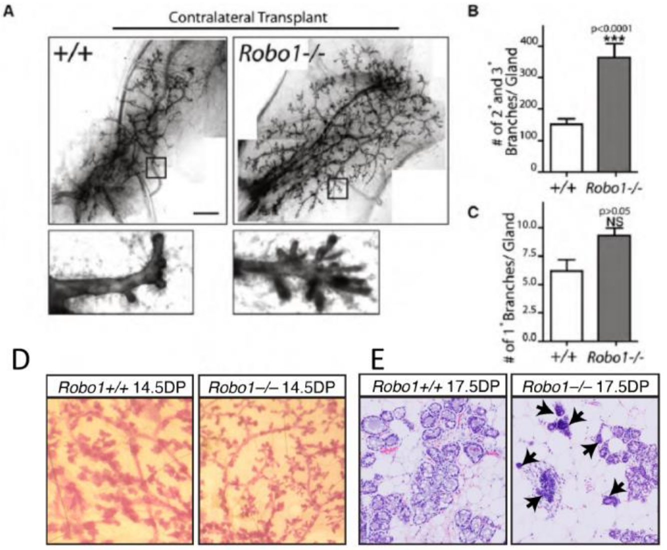

Figure 5: (A-C from Macias et al., D unpublished, E is Figure 1D in current manuscript): (A-C) Contralaterally transplanted, hematoxylin-stained, virgin WT and *Robo1-/-* outgrowths show exuberant branching in the KO glands (A). Quantification of 2°/3° (B) and 1 ° branches/gland (C). **(D)** Whole mounts of *Robo1+/+* and *Robo1-/-* mammary gland tissue at 14.5 days pregnant (DP). (**E**) Images from the current manuscript (Fig. 1E) showing H&E stained and sectioned *Robo1+/+* and *Robo1-/-* mammary gland tissue with *Robo1-/-* alveoli appearing compact and lumenless.

When we performed the RNAseq experiment, we did indeed analyze the basal and mature luminal subpopulations. All these data are uploaded so that readers can explore them (GSE164707). Below you can see pathway analysis from the Enrichr software that shows changes in canonical ROBO1 signaling pathways (Abl (Bashaw et al., 2000) (Rhee et al., 2007) and Cdc42/Rac (Lundström et al., 2004) (Le et al., 2016)) in *Robo1-/-* basal cells (Figure 6A). These pathways that are likely involved ROBO's mediation of cell/cell and cell/ECM interactions. Here, we also present the Enrichr software analysis for the mature luminal cells that revealed pathways involved in gas exchange and small molecule transport (Figure 6B). While we may pursue these interesting targets in the future, we decided to focus on the alveolar progenitor cells because our analysis indicated reduced differentiation, which matched our phenotypic observations. To make our logic clearer to the reader, we changed the manuscript as follows:

Lines 116-120, *Piquing our interest was the KEGG analysis on LPs that revealed not only pathways consistent with current data on ROBO function, such as ECM-receptor interaction and regulation of actin cytoskeleton (Fig. S1A, B) (Blockus and Chedotal, 2016), but also downregulation of Jak-STAT and prolactin signaling pathways, which could interfere with successful alveologenesis in pregnant Robo1-/- animals. We also observed downregulation of genes involved in the terminal differentiation of alveolar epithelium,…..*

We published the ratio of the basal populations in a previous paper on branching morphogenesis Macias et al. (Figure 6C); we observed an increase in the number of basal cells. The ratios of the luminal populations were included in Supplemental Figure 3C of the submitted manuscript; the data show an increase in AVPs, a non-significant but trending decrease in LPs and no change in MLs (Figure 6D).

© 2021. Published by The Company of Biologists under the terms of the Creative Commons Attribution License (https://creativecommons.org/licenses/by/4.0/).

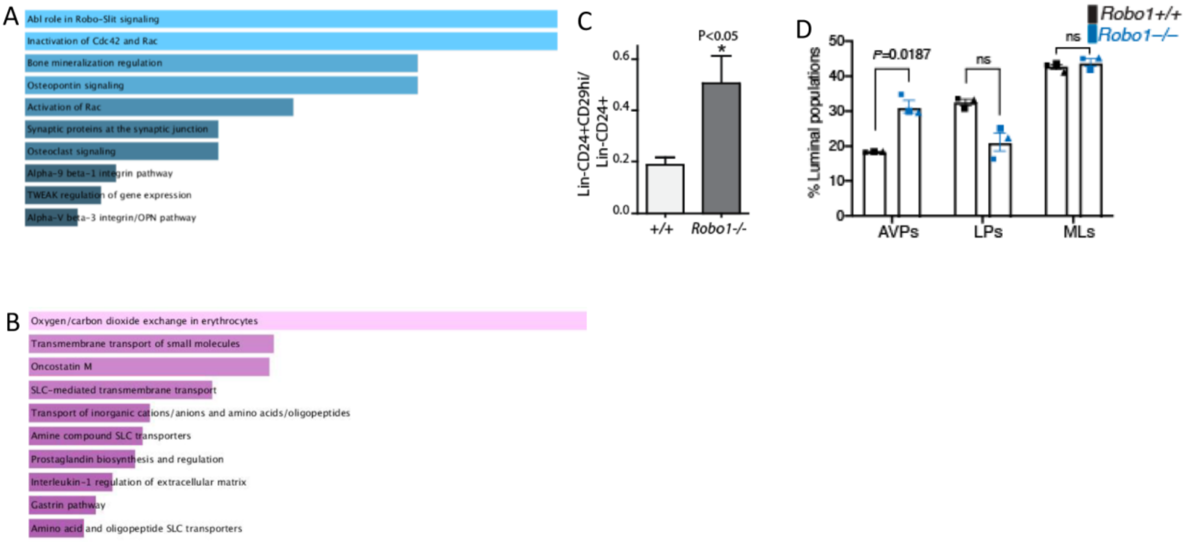

Figure 6: (A, B) Enrichr software analysis identified the pathways that are regulated in FACS-purified *Robo1-/-* basal cells (A) and *Robo1-/-* mature luminal cells (B) that were collected from mature virgin *Robo1+/+* and *Robo1-/-* animals. (C) FACS analysis of the relative level of basal (Lin_CD24+CD29hi) to total (Lin_CD24+) epithelial cells in *Robo1-/-* and *WT* littermate glands (excerpted from (Macias et al., 2011)). (D) (Manuscript Fig. S3C). FACS analysis of *Robo1+/+* and *Robo1-/-* luminal subpopulations

Line 242: MDA-MB-23? Supposedly, MDA-MB-231 cells here? These are not basal mammary cells. MCF10A cells are a good model for basal mammary epithelial cells and should be used.

Because MDA-MB-231 cells are not the ideal cell line to evaluate (as per the Reviewer's point), we removed the data and rather than examining a different cell line, we instead include the analysis of primary cells in the revised manuscript. We show a Western blot demonstrating increased JAG1, but not JAG2, in FACS-purified populations of *Robo1+/+* and *Robo1-/-* basal cells.

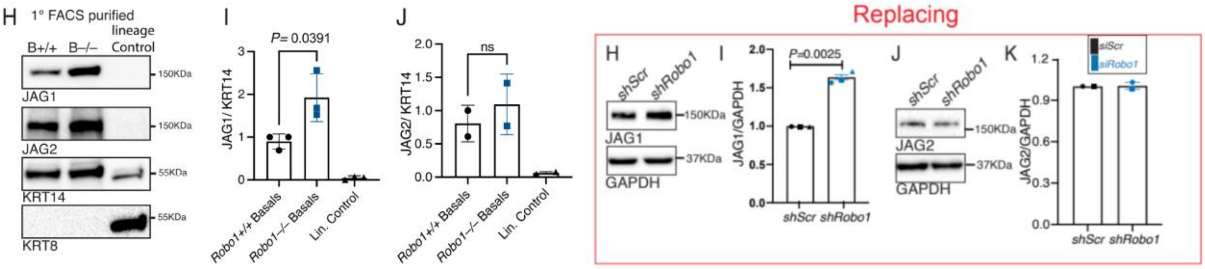

Figure 7: Left (revised Figure S5H-J): Immunoblot (H) and quantification (I, J) show increased JAG1, but not JAG2, in *Robo1-/-* FACS-purified basal cells (two-tailed paired *t*-test). **Right (replaced Figure S5H-J).**

Line 241-242: We also examined JAG expression in FACS-purified populations of Robo1+/+ and Robo1-/- basal cells and found more JAG1 in KO, compared to WT, cells but no significant change in JAG2 (Fig S5H-J).

Line 276/277. The conclusion is an overstatement. The nice in vitro data indicate an important role for jagged1 in lactogenic differentiation as assessed by CSN2 expression. The term alveologenesis only applies to the in vivo phenomenon that physiologically occurs during pregnancy.

We have modified our conclusions to reflect our data in organoids. In the revised manuscript, we conclude:

Lines 276-279: *Altogether, our data suggest that JAG1 is a key regulator of lactogenic differentiation and that downregulation of Jag1 by ROBO1/CTNNB1 in the basal compartment of the organoids inhibits luminal Notch activity, thereby promoting alveolar cell differentiation and CSN2 expression (Fig 6F).*

© 2021. Published by The Company of Biologists under the terms of the Creative Commons Attribution License (https://creativecommons.org/licenses/by/4.0/).

Minor concerns:

1.Running title (and throughout the ms): there are no lobules in the mouse mammary glands should read “mammary alveologenesis”

Thank you for the correction; we have corrected this throughout the manuscript.

2.Abstract: line 46: “Breast” is used only for the human organ here: mammary gland

Corrected

3. Figure 1B is puzzling: DP7.5 (typo??) there is no alveologenesis. “In the developing alveoli” is imprecise, on a section you cannot tell small branches from alveoli. If thick section was used to ensure 3D interpretation, please mention.

In the revised manuscript, we clarify that this image is, indeed, from a thick section of CUBIC-cleared at 7.5DP tissue. We include a Supplemental video and changed the wording from developing alveoli to tertiary buds.

Lines 133-136: *We performed immunohistochemistry (IHC) on thick sections of CUBIC-cleared 7.5DP tissue and observed ROBO1 in subpopulations of myoepithelial and luminal cells of tertiary buds (Fig. 1B), with no expression observed in Robo1-/- tissue (Fig. S1F, G) (Long et al., 2004).*

4. Line 166 “2D organoids” does not make sense, better refer to ability to form domes on plastic.

We removed the term 2D organoid. Below is the revised description.

Line 165-6: *This heterogeneous cell line grows with Keratin 14 (KRT14)-positive basal cells encircling Keratin 8 (KRT8)-positive luminal cells (Fig 2I).*

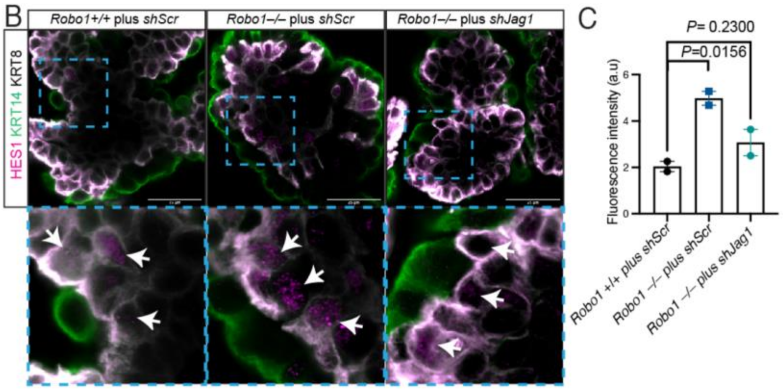

Figure 8 (revised Figure 6B, C): (B, C) Representative 3D confocal images (B) and quantification (C) of *Robo1+/+* and *Robo1-/-* organoids infected with either *shScr* or *shJag1* and stained for HES1 (magenta) and KRT14 (green) show increased HES1 expression in *Robo1-/-* basal cells that is decreased by KD of *Jag1* (*shJag1*).

5. Wnt4 was shown to activate canonical Wnt signaling in the basal epithelium as assessed by axin-2 transcription. This is important for mammary stem cell function but not alveologenesis (Rajaram RD et al EMBO 2015). How may the wnt4 induced Wnt signaling in basal cells interact with the one induced by Robo-1 here?

Thank you for bringing up this interesting point. Indeed Rajaram and colleagues showed, using the Axin-2 reporter (i.e. beta-galactosidase activity) canonical Wnt signaling at day 14.5 of pregnancy in the myoepithelial cells of ducts, but not in the newly formed alveoli. A previous study showed deficient 2^{O} and 3° branching in *Wnt4-/-* epithelium in early pregnancy but ultimately normal development of alveoli (Brisken et al., 2000). Overexpression of *Wnt4* in virgin mammary gland does not induce hyperbranching in virgin mice or during early morphogenesis during pregnancy (Kim et al., 2009). Taken together, this data suggest that *Wnt4* is necessary, but not sufficient, to generate 2^{O} and 3° branches during pregnancy. The general consensus appears to be that WNT4 functions downstream of progesterone, with the hormone upregulating *Wnt4* expression in PR+ mature luminal “sensor” cells. In turn, WNT4 induces canonical signaling in adjacent myoepithelial

© 2021. Published by The Company of Biologists under the terms of the Creative Commons Attribution License (https://creativecommons.org/licenses/by/4.0/).

cells, leading to changes in gene expression (Rajaram et al., 2015).

As to the Reviewer's point -- how WNT4 signaling may interact with the beta-catenin signaling induced by loss of *Robo1* -- we performed RT-qPCR on FACS-purified *WT* and *KO* mature luminal subpopulations (i.e. on the population containing *Wnt4*-expressing "sensor" cells), collected from virgin, PD 10.5 and PD 17.5 mammary glands (Figure 9A). We see no regulation of *Wnt4* mRNA in *Robo1* KO cells at any of these stages of development, suggesting that both *Wnt4* expression and WNT4's functional signaling (to ductal basal cells) is unaffected by ROBO1. Instead, taken together, our studies suggest that ROBO1 downregulation of JAG1 in basal cells negatively affects alveolar differentiation and milk production by inhibiting luminal Notch signaling and promoting differentiation. We see that *Robo1-/-* mammary glands contain more AVPs (Figure 9B and Figure S3C in current manuscript) and increased Notch signaling in those AVPs (Figure 9C and Figure 3H in the current manuscript). Thus we *suggest that by PD7.5 ROBO1-directed repression of nuclear CTNNB1 reduces Jag1 expression in basal MECs, triggering a switch that dials back Notch signaling in adjacent luminal cells to promote the differentiation of AVPs* (lines 339-341). We postulate that *Wnt4*, expressed by mature luminal "sensor" cells affects signaling in adjacent basal cells that comprise a different subpopulation of basal cells than those affected by ROBO1 regulation of beta-catenin, which occurs in another subset of basal cells and influences the expansion or differentiation of luminal AVPs.

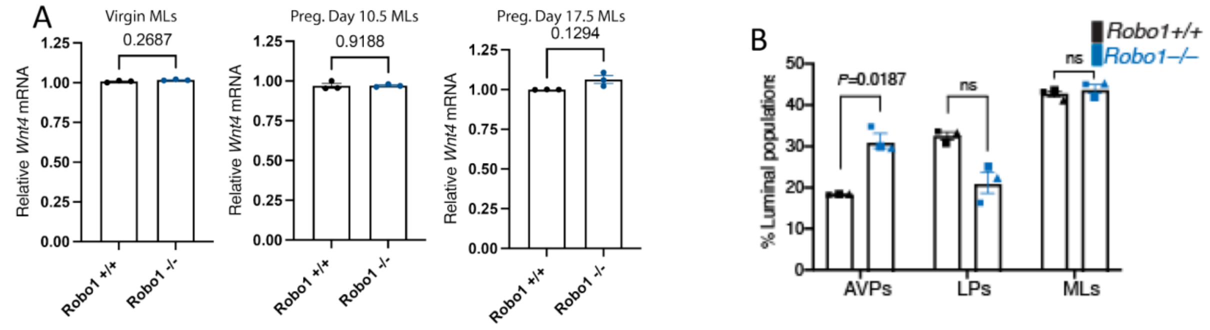

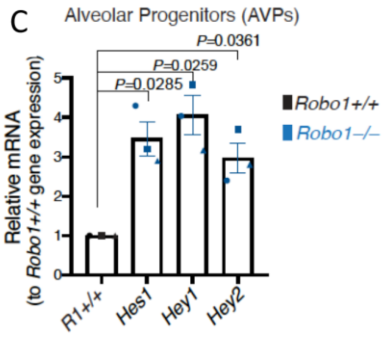

Figure 9: (A) RT-qPCR for Wnt4 on FACS-purified mature luminal cells (LMs) harvested from virgin, Pregnant Day 10.5 and Pregnant Day 17.5 mammary glands. n=3. (B) (current manuscript Figure S3C). FACS quantification of *Robo1*+/+ and *Robo1*-/- luminal subpopulations. *Robo1*-/- MGs contained significantly more AVPs, fewer LPs (trending) and no difference in the number of mature luminal cells (MLs). (C) (current manuscript Figure 3H). RT-qPCR on FACS-purified *Robo1*+/+ and *Robo1*-/- AVPs shows increased Notch effector expression with loss of *Robo1*.

References:

Bashaw, G. J., Kidd, T., Murray, D., Pawson, T. and Goodman, C. S. (2000). Repulsive axon guidance: Abelson and Enabled play opposing roles downstream of the roundabout receptor. *Cell* **101**, 703-715.

Blockus, H. and Chedotal, A. (2016). Slit-Robo signaling. *Development* **143**, 3037-3044.

Brisken, C., Heineman, A., Chavarria, T., Elenbaas, B., Tan, J., Dey, S. K., McMahon, J. A., McMahon, A. P. and Weinberg, R. A. (2000). Essential function of Wnt-4 in mammary gland development downstream of progesterone signaling. *Genes Dev* **14**, 650-654.

Harburg, G., Compton, J., Liu, W., Iwai, N., Zada, S., Marlow, R., Strickland, P., Zeng, Y. A. and Hinck, L. (2014). SLIT/ROBO2 signaling promotes mammary stem cell senescence by inhibiting Wnt signaling. *Stem cell reports* **3**, 385-393.

Kim, Y. C., Clark, R. J., Pelegri, F. and Alexander, C. M. (2009). Wnt4 is not sufficient to induce lobuloalveolar mammary development. *BMC developmental biology* **9**, 55.

Le, L. T., Cazares, O., Mouw, J. K., Chatterjee, S., Macias, H., Moran, A., Ramos, J., Keely, P. J., Weaver, V. M. and Hinck, L. (2016). Loss of miR-203 regulates cell shape and matrix

© 2021. Published by The Company of Biologists under the terms of the Creative Commons Attribution License (https://creativecommons.org/licenses/by/4.0/).

adhesion through ROBO1/Rac/FAK in response to stiffness. *J Cell Biol* **212**, 707-719.
Long, H., Sabatier, C., Ma, L., Plump, A., Yuan, W., Ornitz, D. M., Tamada, A., Murakami, F., Goodman, C. S. and Tessier-Lavigne, M. (2004). Conserved roles for Slit and Robo proteins in midline commissural axon guidance. *Neuron* **42**, 213-223.
Lundström, A., Gallio, M., Englund, C., Steneberg, P., Hemphälä, J., Aspenström, P., Keleman, K., Falileeva, L., Dickson, B. J. and Samakovlis, C. (2004). Vilse, a conserved Rac/Cdc42 GAP mediating Robo repulsion in tracheal cells and axons. *Genes Dev* **18**, 2161-2171.
Macias, H., Moran, A., Samara, Y., Moreno, M., Compton, J. E., Harburg, G., Strickland, P. and Hinck, L. (2011). SLIT/ROBO1 signaling suppresses mammary branching morphogenesis by limiting basal cell number. *Dev Cell* **20**, 827-840.
Rajaram, R. D., Buric, D., Caikovski, M., Ayyanan, A., Rougemont, J., Shan, J., Vainio, S. J., Yalcin-Ozuysal, O. and Brisken, C. (2015). Progesterone and Wnt4 control mammary stem cells via myoepithelial crosstalk. *Embo j* **34**, 641-652.
Rhee, J., Buchan, T., Zukerberg, L., Lilien, J. and Balsamo, J. (2007). Cables links Robo-bound Abl kinase to N-cadherin-bound beta-catenin to mediate Slit-induced modulation of adhesion and transcription. *Nat Cell Biol* **9**, 883-892.

Second decision letter

MS ID#: DEVELOP/2021/199940

MS TITLE: Alveolar Progenitor Differentiation and Lactation Depends on Paracrine Inhibition of Notch via ROBO1/CTNNB1/JAG1

AUTHORS: Oscar E Cazares, Shamila E Chatterjee, Pinky Lee, Catherine E Strietzel, J W Bubolz, Gwyndolen E Harburg, Jon E Howard, Sol E Katzman, Jeremy E Sanford, and Lindsay E Hinck
ARTICLE TYPE: Research Article

I am happy to tell you that your manuscript has been accepted for publication in Development, pending our standard ethics checks.

Reviewer 1

Advance summary and potential significance to field

The studies describes in this manuscript focus on identification of a regulatory pathway important for regulating alveolar differentiation and lactation. This pathway involves ROBO1 as a key regulator of Notch signaling, and the studies delineate the specific signaling mechanism involved that contribute to regulation of alveolar differentiation. These studies provide new information regarding the mechanisms driving alveolar differentiation, relevant to the mammary gland development field and the more general field of epithelial differentiation.

Comments for the author

The authors have responded thoroughly to my comments. I have no further concerns.

Reviewer 2

Advance summary and potential significance to field

Cazares et al demonstrate that loss of Robo1 in the mammary gland epithelium results in activation of Notch signaling which leads to an expansion of alveolar progenitor cells and impairs alveolar differentiation. Robo1 inhibits the expression of Notch ligand Jagged-1 by regulating ß-catenin in basal/myopepithelial cells.

© 2021. Published by The Company of Biologists under the terms of the Creative Commons Attribution License (https://creativecommons.org/licenses/by/4.0/).

This work provides new insights into the communication between different cell types in the mammary epithelium that are important in controlling its functional differentiation.

Comments for the author

The authors have adequately addressed my concerns.

© 2021. Published by The Company of Biologists under the terms of the Creative Commons Attribution License (https://creativecommons.org/licenses/by/4.0/).
